# *MIDAS*: a quantitative framework for high-energy diffraction microscopy. Part II: accuracy, robustness and best practices

**DOI:** 10.1107/S2053273326004018

**Published:** 2026-05-28

**Authors:** Hemant Sharma, Jun-Sang Park, Sarvjit Shastri, Peter Kenesei

**Affiliations:** a Advanced Photon Source, Argonne National Laboratory, 9700 S. Cass Ave., Lemont, IL 60439, USA; Institute of Crystallography - CNR, Bari, Italy

**Keywords:** HEDM, high-energy diffraction microscopy, 3DXRD, 3D X-ray diffraction, *MIDAS*, experimental validation, performance analysis

## Abstract

The increasing complexity of *in situ* high-energy diffraction microscopy (HEDM) experiments demands a quantitative understanding of the data analysis pipeline to ensure reproducible science. However, the influence of key analysis parameters on the accuracy and precision of microstructural reconstructions is often not well quantified, creating a barrier to progress. This paper addresses this critical gap by presenting a rigorous, systematic validation of the HEDM data reduction methodology as implemented in the *MIDAS* software suite. Using a new, dedicated Ti-7 Al dataset, we investigate both far-field (FF) and near-field (NF) HEDM. Our results reveal critical sensitivities, demonstrating that grain position accuracy in FF-HEDM is highly dependent on the diversity of sampled diffraction vectors, while orientation precision in NF-HEDM improves dramatically with increased detector separation. We demonstrate the methodology’s robustness against common experimental challenges, such as severe diffraction peak overlap, which is effectively filtered by requiring crystallographic consistency. Based on these quantitative findings, we establish a framework of best practices for HEDM data acquisition and analysis to guide the community towards more accurate and reliable results.

## Abstract 2

This paper experimentally establishes the accuracy, robustness and performance limits of the high-energy diffraction microscopy data reduction methodology. Using dedicated far-field and near-field datasets, it quantifies the influence of key analysis parameters, demonstrates computational efficiency, and establishes a framework of best practices to guide the community towards more reproducible results.

## 1. Introduction

High-energy diffraction microscopy (HEDM) is a suite of diffraction-based imaging techniques utilizing high-energy X-rays capable of non-destructively characterizing polycrystalline materials at the micrometre scale. Two dominant HEDM techniques, far-field (FF) and near-field (NF) HEDM techniques, have been successfully applied to study microstructural changes in materials under different types of stimulus. Examples include deformation in structural alloys (Park *et al.*, 2015 ▸; Naragani *et al.*, 2019 ▸; Naragani *et al.*, 2017 ▸; Sangid *et al.*, 2018 ▸; Paranjape *et al.*, 2017 ▸; Paranjape *et al.*, 2018 ▸; Zhang *et al.*, 2018 ▸), grain growth during thermal treatment (Sharma *et al.*, 2016 ▸; Sharma *et al.*, 2012 ▸), load partitioning and transfer in geological materials (Amirrahmat *et al.*, 2020 ▸; Imseeh *et al.*, 2020 ▸; Zhai *et al.*, 2020 ▸), and energy materials (Dixit *et al.*, 2022 ▸) to name a few.

The primary motivation for this work is to provide a rigorous, quantitative foundation for HEDM data analysis, moving beyond anecdotal performance reports. While the methodological framework has been successfully employed (as implemented in the *MIDAS* software) as a tool in prior materials science studies (Amirrahmat *et al.*, 2020 ▸; Yan *et al.*, 2017 ▸; Yan *et al.*, 2018 ▸; Sangid *et al.*, 2018 ▸; Paranjape *et al.*, 2017 ▸; Paranjape *et al.*, 2018 ▸; Imseeh *et al.*, 2020 ▸; Naragani *et al.*, 2017 ▸; Naragani *et al.*, 2019 ▸; Park *et al.*, 2015 ▸; Zhang *et al.*, 2018 ▸), those studies focused on materials-specific outcomes. The present work has a fundamentally different and more foundational goal: to scientifically investigate the data analysis methodology itself. This paper is therefore not a demonstration of the software, but an investigation into the stability, accuracy and sensitivity of the HEDM data reduction process.

The specific objectives of this paper are to:

(i) Quantify the accuracy and precision of both the FF- and NF-HEDM reconstruction pipelines using new, dedicated experimental data.

(ii) Systematically evaluate the influence of critical user-defined analysis parameters (*e.g.* choice of indexing rings, confidence thresholds, detector separation) on final reconstruction quality.

(iii) Demonstrate the methodology’s intrinsic robustness against common experimental challenges, such as severe diffraction peak overlap and minor scan-to-scan misalignments.

(iv) Establish a clear, evidence-based framework of best practices for HEDM data acquisition and analysis to guide the community.

It is important to differentiate this work from the recent Sparks *et al.* (2024 ▸) publication. The Sparks paper utilized HEDM data to compare them with electron backscatter diffraction (EBSD). This paper, by contrast, uses a new, unpublished Ti-7 Al dataset to conduct an introspective analysis of the HEDM methodology itself. Furthermore, where the Sparks paper briefly noted analysis challenges, this work uses its final section to delve deeply into those limitations, using the public Sparks dataset as a case study for future methodological development.

## 2. FF-HEDM

### 2.1. Experimental details

A sample of Ti-7 Al with 1 × 1 mm cross section was chosen for the experimental study. The sample was placed in the RAMS device (Shade *et al.*, 2015 ▸) and illuminated by a vertically focused X-ray beam [monochromated (Shastri *et al.*, 2002 ▸; Shastri, 2004 ▸) with *E* = 65.351 keV, Hf *K* edge (Hubbell & Seltzer, 2024 ▸)] with a full width at half-maximum (FWHM) of 0.9 µm [vertical, focused using a sawtooth lens (Shastri *et al.*, 2020 ▸)] and 2.0 mm (horizontal, defined using slits). For the FF-HEDM experiment, a GE-41RT detector with pixel pitch of 200 µm and 2048 × 2048 pixels (Lee *et al.*, 2008 ▸) was placed at a distance of approximately 940 mm from the sample and diffraction data were collected by continuously rotating the sample over 360° and acquiring data at 0.25° intervals with 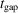
 = 0.126 s and 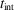 = 0.02 s. [For details about 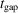 and 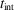, readers are directed to Part I of the series (Sharma *et al.*, 2026 ▸).] The dynamic range of the detector pixels is 14-bit. The rings with the smallest 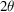 angles result in the highest diffracted intensities. (Apart from variations due to the structure factor, the intensity of a peak decreases with 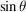.) Thus, a disc-shaped attenuator was placed in the center of the detector to improve the diffraction signal from diffraction rings with the highest 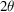, while reducing the signal on the first three rings of the sample. The smaller 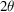 rings also have the worst strain sensitivity, which increases linearly with 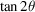. A CeO_2_ powder was used to determine the parameters of the experimental setup, and the sample itself was used to determine 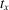 and Ω angles. The refined parameters are given in Table 1 ▸. The mean strain observed after fitting the CeO_2_ powder pattern was 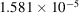. The setup was aligned to ensure Ω was below the detection limit (<0.02°).

### 2.2. Data analysis

The analysis, implementing the FF-HEDM methodology from Part I (Sharma *et al.*, 2026 ▸), was run on a high-performance cluster (HPC) using five nodes, each with 128 AMD EPYC CPU cores. Demonstrating the computational efficiency of the framework, each reconstruction took about 2 min to finish. The reference crystal parameters used are given in Table 2 ▸. The attenuator reduces the intensity of diffraction peaks for the first three rings, and the attenuation of the diffraction signal from the attenuator is not uniform. Thus, diffraction rings 1–3 were not included in the analysis. Table 3 ▸ shows the characteristics of diffraction rings for the Ti-7 Al sample used in the present analysis. After subtracting a dark image (image acquired without any X-rays) from diffraction patterns to remove electronic noise, a threshold of 80 counts was applied to detect peaks. A tolerance of 15 pixels (3000 µm) from the ideal ring radii was used to define the pixels with a diffraction signal. As seen in Table 3 ▸, the ring radii for rings 8 and 9 (highlighted in bold) are separated by less than the tolerance (3000 µm), leading to multiple peaks from either ring being assigned to the other.


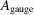
 for grain size calculation was calculated from sample dimensions using microtomography (µ-CT) results. [The framework has an in-built µ-CT reconstruction module, which uses GridRec (Marone & Stampanoni, 2012 ▸) for reconstruction.] Only diffraction peaks with equivalent grain radius within 5% of each other were indexed together. For the reference reconstruction, diffraction peaks on rings 4–15 (RingsToAnalyze = [4–15]) were used. Diffraction peaks on ring 5 (RingToIndex = {110}) with 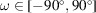 were used to generate candidate orientations, *MinNrSols* was set to 3,^1^ and only grain candidates with *Confidence* > 0.2 were qualified as grains. A virtual cylinder of radius 1800 µm and height 800 µm was used for constraining the reconstruction space. The number of expected peaks is 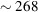, so a confidence of 0.2 means at least 54 diffraction peaks were indexed to the grain.

The parameters described in Part I of the series were systematically varied to evaluate the framework’s performance, demonstrate its robustness and establish best practices. Their effect on the results is presented next. For reference, these parameters are:

(i) *RingsToAnalyze*: diffraction rings to use for analysis.

(ii) *RingToIndex*: diffraction ring to use for generation of candidate orientations.

(iii) *MinNrSols*: minimum number of times the same solution needs to be found for an orientation to be valid.

(iv) *Confidence*: the minimum ratio of observed versus expected number of diffraction peaks for an orientation to be valid.

(v) *LatticeParameter*: reference lattice parameter for indexing and strain calculation.

Readers are directed to Part I of this series for a detailed description of the parameters.

The Ti-7 Al dataset used in this study was acquired at Advanced Photon Source (APS) beamline 1-ID-E prior to the APS-U upgrade. It therefore represents a reference measurement of HEDM achievable accuracy at the pre-upgrade source, and serves as a baseline for future comparisons with post-upgrade measurements that benefit from increased brilliance, reduced horizontal emittance and new detector hardware. The raw data have been deposited in the Materials Data Facility (see *Data availability*), making this baseline accessible to the community for cross-validation with alternative HEDM software packages and for benchmarking future methodological improvements.

### 2.3. Results and discussion

#### 2.3.1. Reference reconstruction

**Figure 1:**
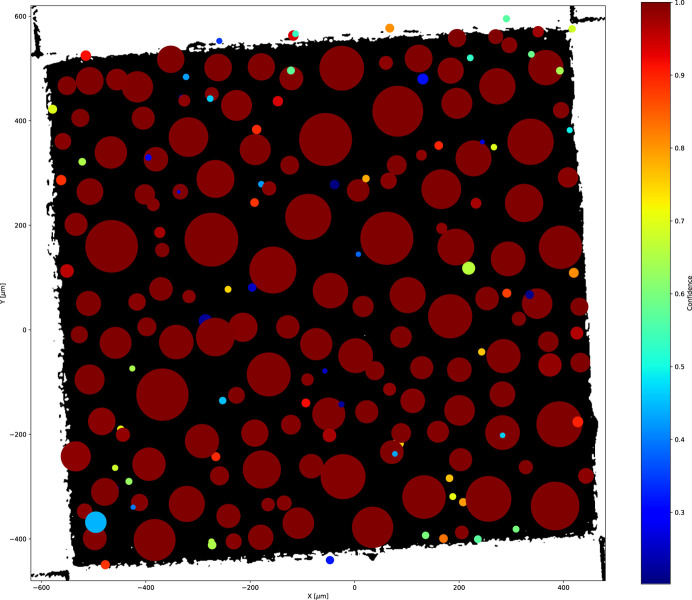
Reference FF-HEDM reconstruction. The position of the grains determined in FF-HEDM is overlaid on tomography reconstruction (in black). Each marker represents a grain’s position, the marker’s size is directly proportional to the grain’s size, and the marker’s color represents Confidence. Black areas outside the sample on the edges are tomography reconstruction artifacts.

**Figure 2:**
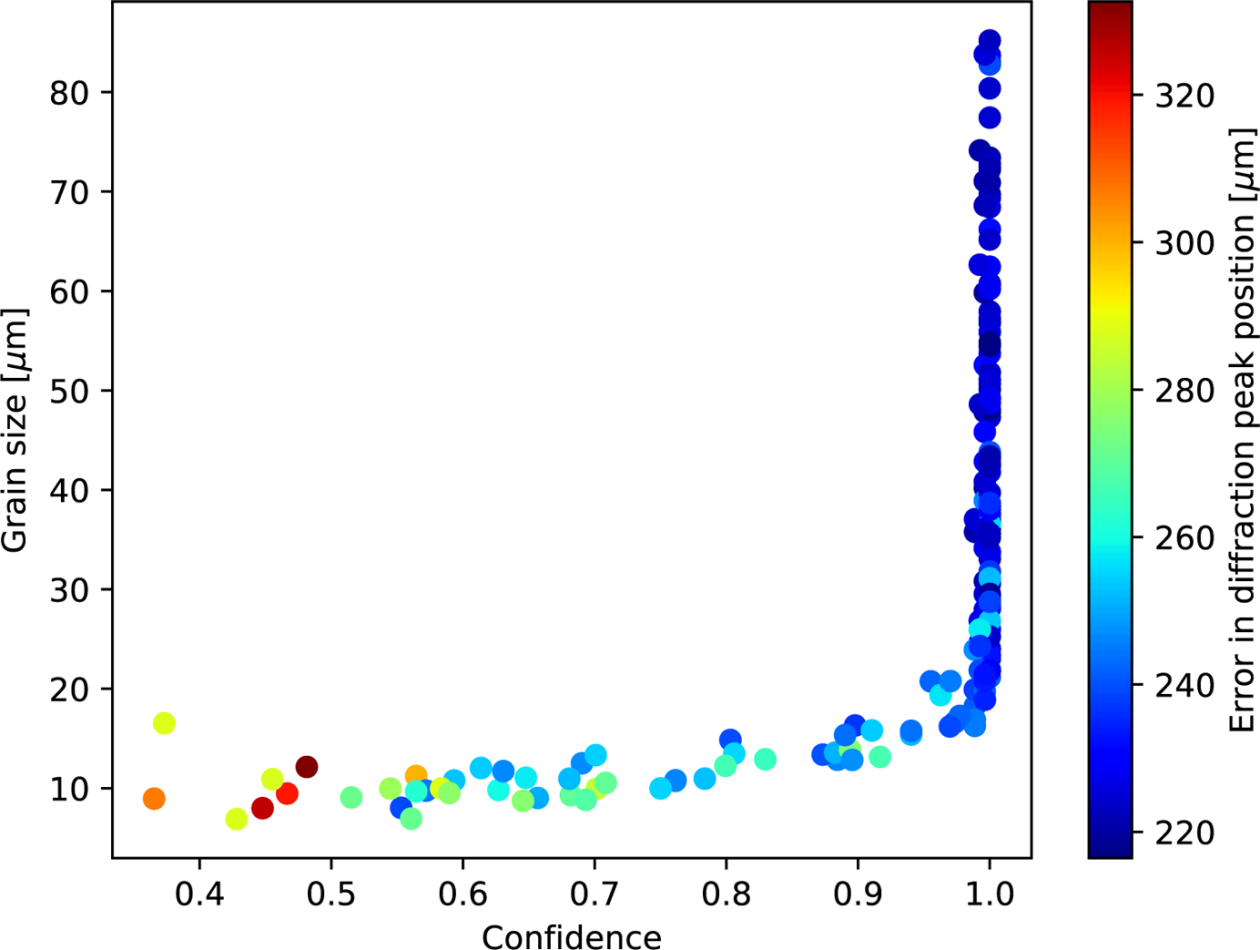
FF_HEDM: grain size as a function of confidence. Markers are colored according to errors in diffraction peak position.

A total of 209 grains were identified in the reference reconstruction. Fig. 1 ▸ shows the position of those grains. The size of the markers in Fig. 1 ▸ is directly proportional to the grain radius. The total area of the grains equals 95.57% of the sample area. It can be seen that most of the grains lie inside the sample. The mean grain radius for this reference reconstruction was determined to be 38 µm, providing context for subsequent analysis. The grains outside the sample all have lower confidence. From Fig. 2 ▸ it can be seen that 144 grains have confidence greater than 0.9. It can also be seen that all the grains with lower confidence are smaller than 20 µm in size. Fig. 2 ▸ also reveals a correlation between lower confidence (smaller marker size) and increased positional error (marker color), suggesting that the Confidence metric, derived from the completeness of the diffraction pattern match (Part I, Section 3.4), serves as a useful indicator of the overall reliability of the refined parameters for a given grain. Smaller grains have weaker diffraction peaks, which makes it difficult to detect all the diffraction peaks reliably. The error in the position of diffraction peaks, indicated by the color of the markers in Fig. 2 ▸, increases as the confidence decreases due to a reduction in the number of diffraction peaks contributing to the grains.

**Figure 3:**
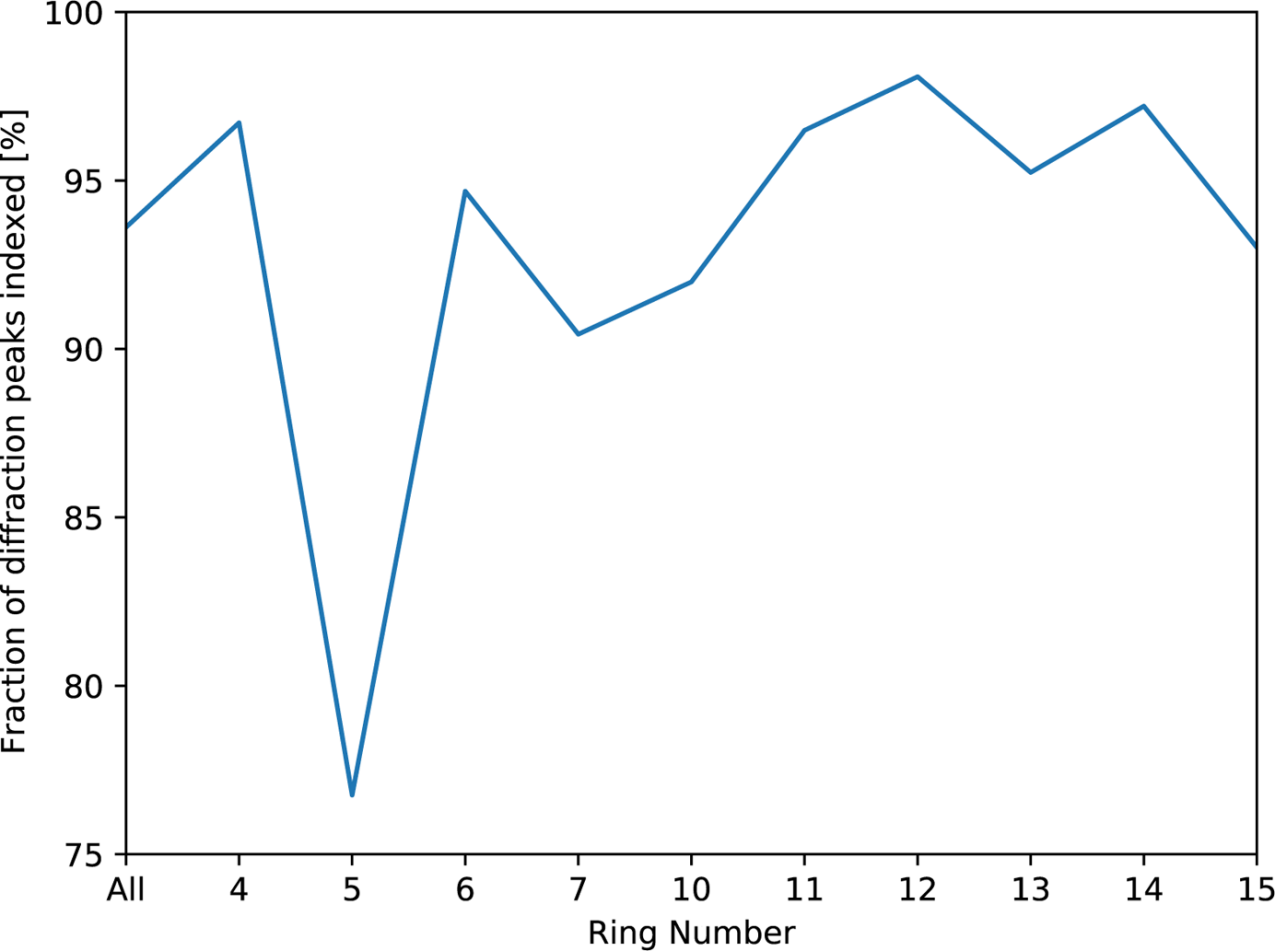
FF_HEDM: fraction of diffraction peaks (%) indexed as a function of the diffraction ring.

In addition to the volume recovery (95.57%), it is interesting to look at the fraction of diffraction peaks that were indexed or assigned to the grains. Fig. 3 ▸ shows the fraction of diffraction peaks indexed as a function of the ring number. Due to the abovementioned problem with peaks being assigned interchangeably to rings 8 and 9, those are excluded from this analysis and dealt with later. 93.6% of the peaks were indexed for all the rings, but it should be noted here that for ring 5, only 76.8% of the peaks were indexed. This is because, as shown in Table 3 ▸, both the theoretical number of grains (calculated by dividing the number of diffraction peaks by 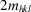) and 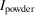 for ring 5 are much higher than those for the other rings. Table 3 ▸ shows a wide spread in 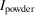 between the rings and from equation (1), 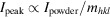; therefore, diffraction signal from the same grain has very different intensities between different rings: diffraction signal from smaller grains is present in ring 5, but not in the other rings. Due to the limited dynamic range of the detector, such a significant change in 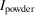 results in a diffraction signal from smaller grains being absent on rings with lower 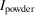. The lower percentage of peaks indexed on ring 5 (76.8%) compared with its high intensity and theoretical grain count (Table 3 ▸) highlights the impact of the large 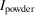 variation. While ring 5 contains signals from many small grains absent on other rings, only a fraction of those specific peaks might be sufficient (along with peaks from other rings for the same grain) to satisfy the Confidence and MinNrSols criteria for successful indexing. Nonetheless, its high overall intensity ensures it provides the initial seeds for finding the most grains, including small ones. 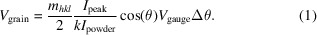
Out of 56543 diffraction peaks belonging to rings 4–15 (excluding rings 8–9), 1003 diffraction peaks were assigned to multiple grains: 980 to two grains and 23 to three grains. For all the peaks assigned to multiple grains, the grain sizes calculated from the peaks were within 5% of the average size calculated from all the peaks of the respective grains.

#### 2.3.2. Probing the framework’s sensitivity and robustness

**Figure 4:**
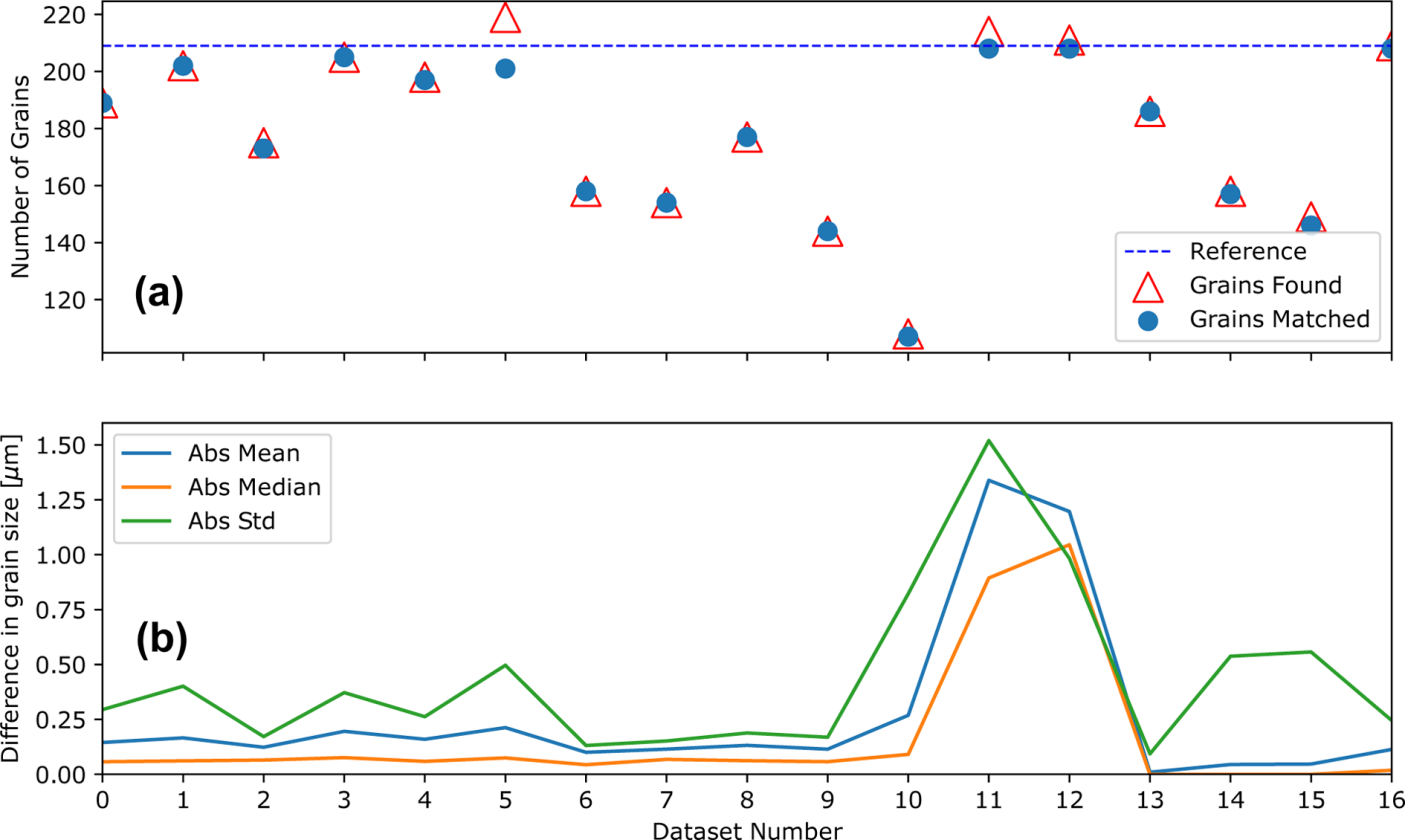
FF_HEDM: results of grain matching for different datasets with reference reconstruction. (*a*) Number of grains found and number of grains matched with the reference for each dataset in Table 4 ▸. (*b*) Difference in grain size between the grains found for the datasets in Table 4 ▸ and reference reconstruction: absolute values for mean (blue), median (orange) and standard deviation (green) lines.

To evaluate the robustness of the framework’s FF-HEDM workflow and guide users on parameter selection, the key analysis parameters were varied systematically from the reference reconstruction, as detailed in Table 4 ▸. A matching strategy introduced by Park *et al.* (2021 ▸) was used to compare the results, calculating a dimensionless parameter, *MatchingCriterion*, based on misorientation and position difference [equation (2)]. The MatchingCriterion [equation (2)] provides a single metric for comparing grain solutions between datasets. Using 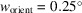 and 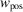 = 50 µm implicitly weights orientation and position differences relative to typical experimental uncertainties or feature sizes. We observed that moderate changes (0.2–2×) to these weights did not significantly alter the matching outcome, which further suggests the stability of the core grain identification. Pairs exceeding 0.6° and 200 µm were filtered out. The results are presented in Figs. 4 ▸ ▸ ▸ ▸ to 8 ▸ and Tables 5 ▸ and 6 ▸. 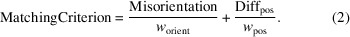


*Sensitivity to initial seeding and reconstruction completeness*. Changing the ring used for initial orientation generation significantly affected the number of grains found [Fig. 4 ▸(*a*)], primarily due to the large intensity variations between rings (Table 3 ▸). This highlights a nuance of FF-HEDM analysis: the choice of indexing ring affects sensitivity, especially to small grains. Ring 5, being the most intense (and thus providing the best signal-to-noise ratio for weak scatterers), yielded the highest probability of finding small grains (evident when comparing datasets 3 and 5, rings 8 versus 10). However, a key strength of the framework is its flexibility and robustness; although grain counts varied, nearly all grains found across different RingToIndex values were correctly matched to the reference reconstruction [Fig. 4 ▸(*a*)]. Best practice: for maximizing completeness, especially if small grains (<20 µm) are critical, using the most intense, complete ring available (like ring 5 here) for RingToIndex is recommended. The framework’s MinNrSols parameter, linked to ring multiplicity (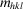), provides a filter against false positives (*e.g.* dataset 5 required reducing MinNrSols to 2 for optimal results when using ring 10 with 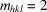).

**Figure 5:**
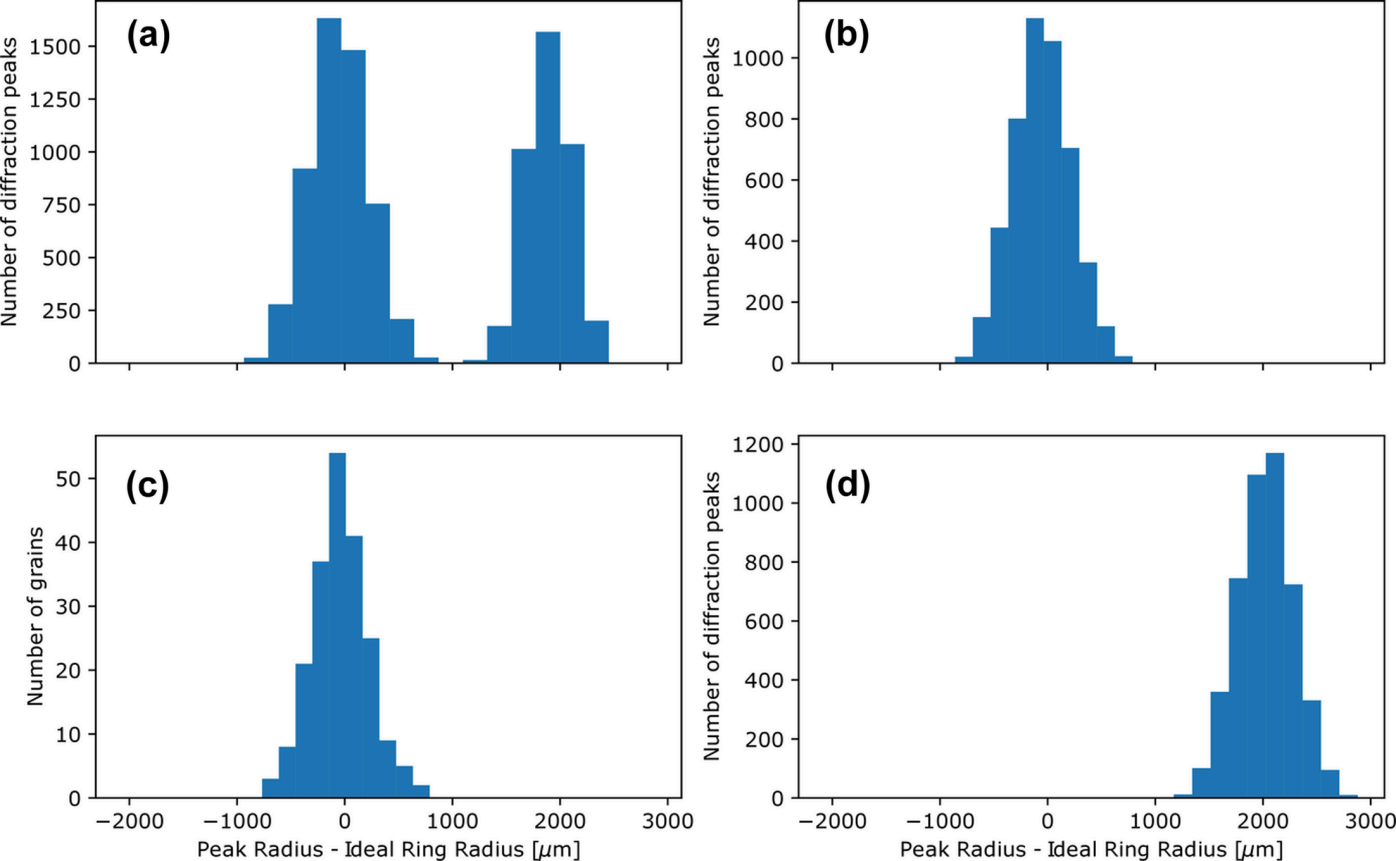
FF_HEDM: diffraction peak analysis of rings 8–9. As a function of radius of each peak position – ideal ring radius of ring 8 (145167.454 µm): (*a*) histogram of diffraction peaks assigned to ring 8, (*b*) histogram of diffraction peaks resulting in grains using ring 8 as RingToIndex, (*c*) histogram of indexed peaks assigned to ring 8 and (*d*) histogram of indexed peaks assigned to ring 9.

*Intrinsic robustness to ambiguous diffraction signals*. The close spacing of rings 8 and 9 led to peaks being assigned to both rings initially [Fig. 5 ▸(*a*)]. This scenario demonstrates a critical strength of the framework: its robustness to such initial misassignments. This robustness arises because the indexing and refinement procedures (Part I, Sections 3.4 and 3.5) require crystallographic consistency across multiple diffraction peaks assigned to a candidate grain. When ring 8 was used for indexing (dataset 3), none of the misassigned peaks belonging to ring 9 could form part of a consistent crystallographic solution for a grain based on ring 8 reflections, and thus resulted in false grain identification [Fig. 5 ▸(*b*)]. Furthermore, the final indexed peak list for ring 8 contained only correctly assigned peaks [Fig. 5 ▸(*c*)], and similarly for ring 9 [Fig. 5 ▸(*d*)]. The framework’s indexing and refinement process effectively filters these initial ambiguities.

*The foundational role of **g** diversity in positional accuracy*. Reducing the number of rings used in the analysis (dataset 11: rings 4–9; dataset 12: rings 4–12 versus reference: rings 4–15) led to a 15% increase in the median position error and a 12% increase in grain size standard deviation, revealing a significant loss of precision [Figs. 4 ▸(*b*), 6 ▸(*c*)–6 ▸(*f*)] and strain (Fig. 7 ▸, Table 5 ▸). This reveals an important nuance: including higher-angle diffraction rings, even if they have lower intensity, improves the accuracy of the grain parameter refinement, particularly for position and strain. This is because accurate determination of grain position relies on triangulating multiple diffraction vectors, and determination of the full strain tensor (Part I, Section 3.7) requires sampling diffraction vectors (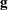
’s, defined as the vector normal to the diffracting crystallographic planes, with a magnitude of 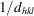) with diverse orientations relative to the crystal lattice to fully constrain tensor components. Excluding higher-angle rings reduces the diversity of the sampled 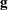
’s, leading to poorer constraints during refinement. A strength of the framework is its ability to efficiently incorporate data from numerous rings. Best practice: include as many reliable diffraction rings as practical in RingsToAnalyze, especially higher-angle ones, to maximize the accuracy of refined grain position and strain, even if a lower-angle ring is used for RingToIndex.

**Figure 6:**
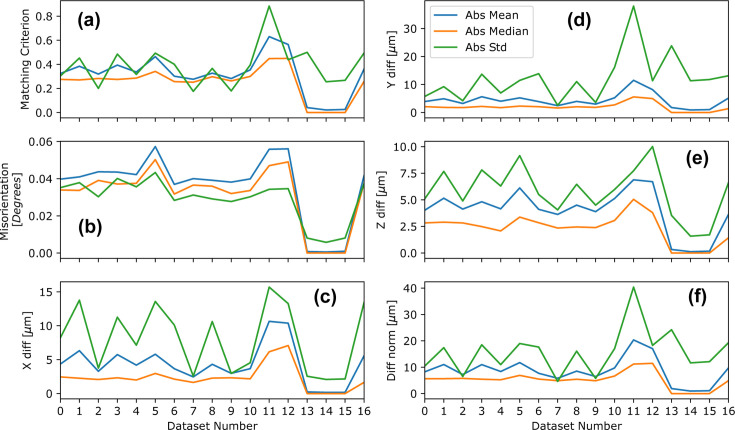
FF_HEDM: error characteristics for grains in reference reconstruction matched with grains found in each dataset in Table 4 ▸: absolute values for mean (blue), median (orange) and standard deviation (green) lines. (*a*) Matching criterion. (*b*) Misorientation angle. Difference in position in (*c*) *X*, (*d*) *Y*, (*e*) *Z*, (*f*) Euclidean distance. The matching criterion is defined in equation (2).

**Figure 7:**
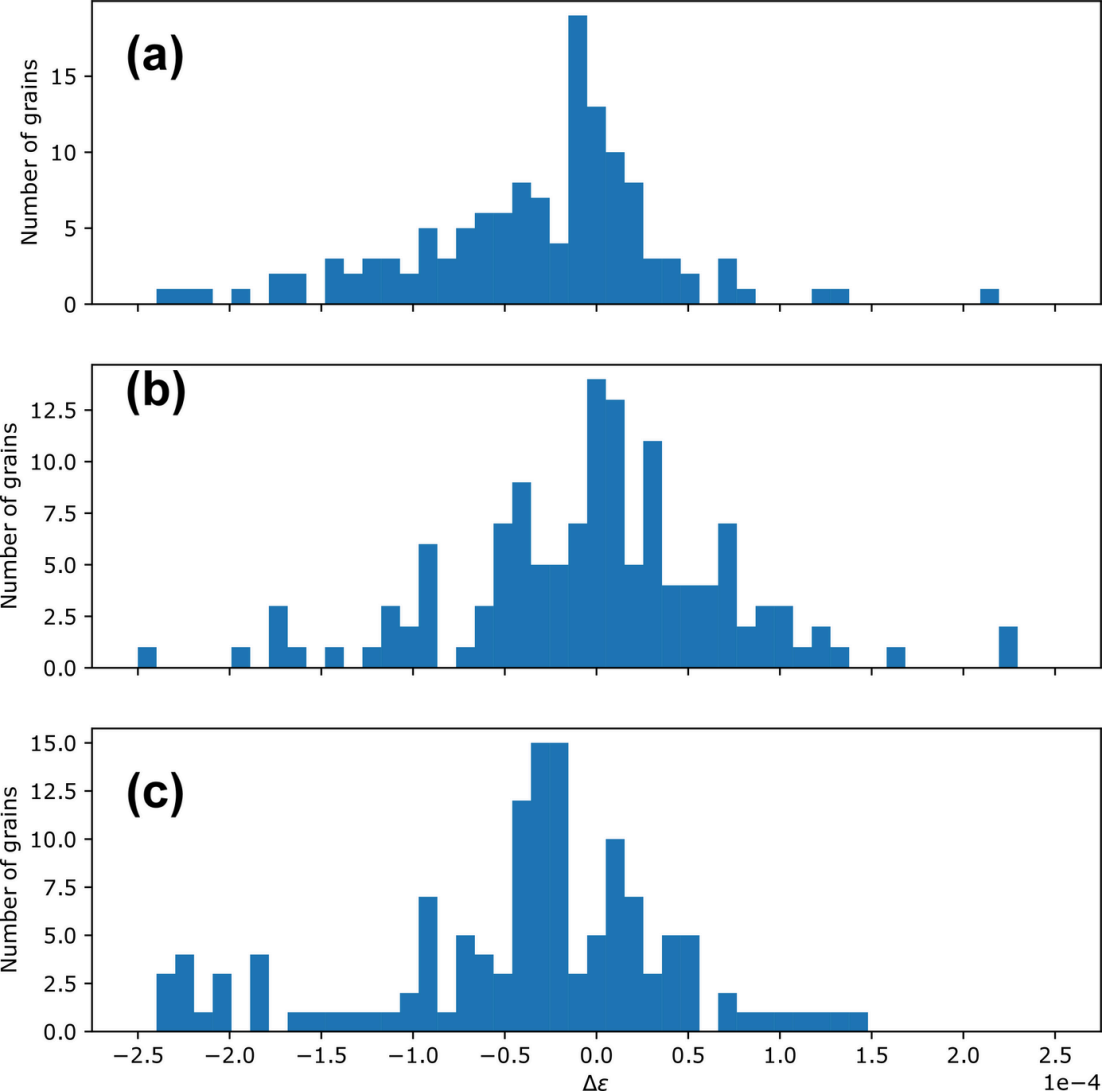
FF_HEDM: histogram of difference in strains between dataset 11 in Table 4 ▸ and reference reconstruction. (*a*) 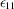, (*b*) 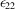 and (*c*) 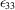.

*Stability of solutions across confidence thresholds*. Varying the Confidence threshold primarily affected the detection of smaller, less complete grains, as expected (Fig. 2 ▸). However, the median and mean errors in orientation, position (Fig. 6 ▸) and grain size (Fig. 4 ▸) remained largely unchanged. This demonstrates the framework’s robustness: the core grain identification is stable across reasonable confidence thresholds, allowing users to tune this parameter based on whether maximizing completeness (lower confidence) or certainty (higher confidence) is prioritized, without drastically altering the results for well defined grains. Best practice: a starting Confidence of 0.2–0.3 is often suitable, adjustable based on data quality and scientific goals.

**Figure 8:**
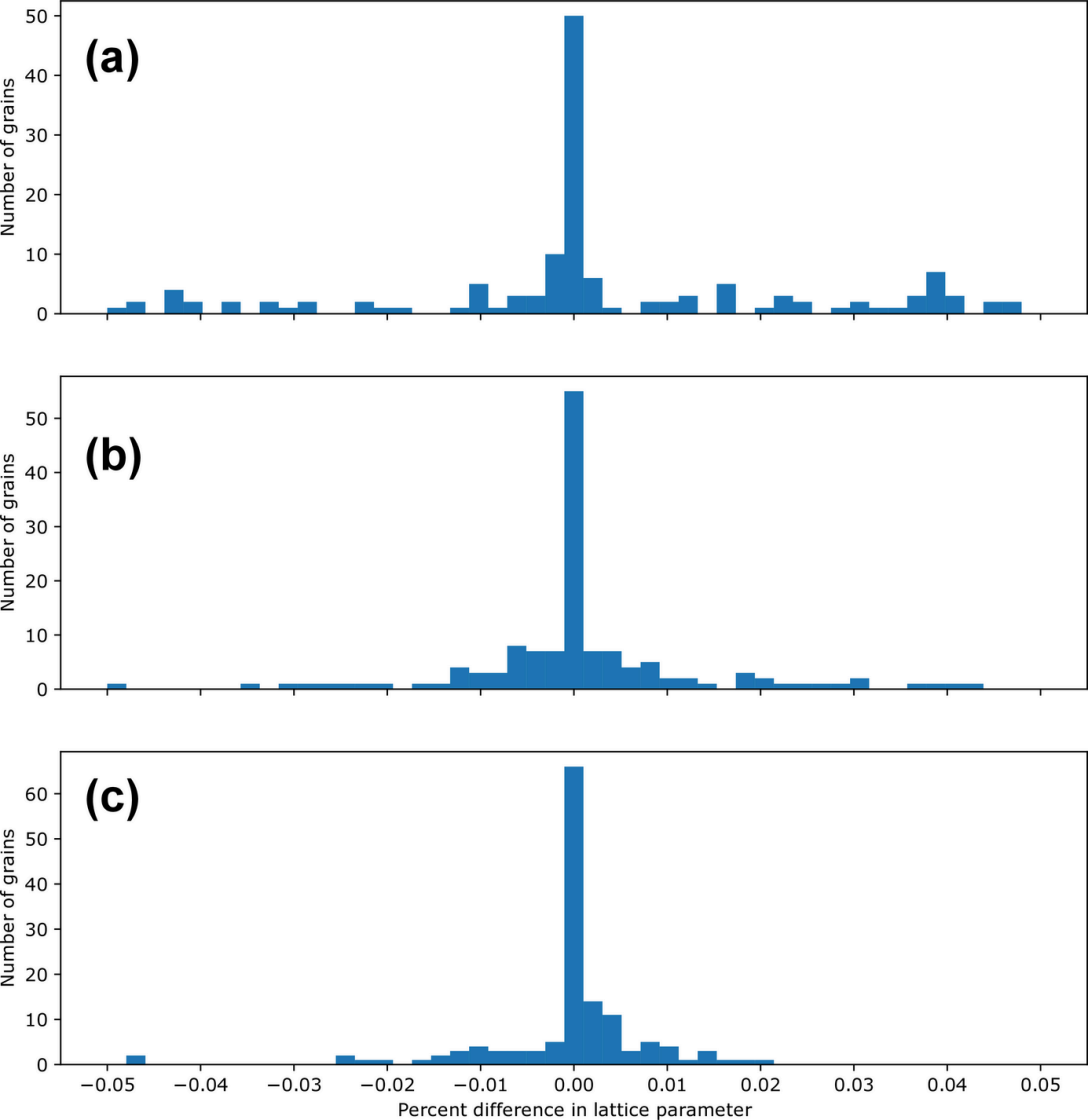
FF_HEDM: histogram of % difference in lattice parameter between dataset 16 in Table 4 ▸ and reference reconstruction. (*a*) *a*, (*b*) *b* and (*c*) *c*.

*Effect of reference LatticeParameter*. Introducing a deliberate initial offset in the lattice parameter used for indexing was correctly handled by the framework. The refined lattice parameters showed median errors smaller than the input deviation (Fig. 8 ▸, Table 6 ▸). This validates the strength and accuracy of the framework’s refinement procedure (Part I, Section 3.5), crucial for obtaining reliable elastic strain measurements.

Overall FF-HEDM validation: the parameter study demonstrates that the framework provides robust and accurate FF-HEDM reconstructions. Please refer to Table 7 ▸ for a summary. While parameter choices influence sensitivity (*e.g.* finding small grains) and precision (*e.g.* position/strain errors), the core grain identification is stable. The key recommendation for optimal accuracy is to include higher-angle diffraction rings in the analysis. The 2 min reconstruction time per dataset on moderate HPC resources further underscores the framework’s efficiency for practical high-throughput studies. The observed median errors in orientation (∼0.05°) and position (∼10 µm) (Fig. 6 ▸) are consistent with previously reported instrument repeatability (Park *et al.*, 2021 ▸), validating the analysis pipeline.

#### 2.3.3. Quantitative validation of the decoupled iterative refinement strategy

A key methodological contribution described in Part I (Section 3.5) is the decoupled iterative refinement scheme, which sequentially optimizes grain position, orientation and lattice parameters using specialized objective functions matched to the differing sensitivities of each parameter group. To quantitatively validate this approach against the conventional alternative – simultaneous optimization of all 12 parameters using a single objective function – we conducted a controlled experiment using synthetic data with known ground truth.

*Simulation setup*. A polycrystalline aggregate of 250 randomly oriented face-centered cubic (f.c.c.) grains (Au, 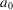 = 4.08 Å, space group 225) was generated with random positions within a cylindrical sample volume (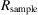 = 2000 µm, 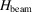 = 2000 µm). Each grain was assigned lattice parameters with random deviations of up to 0.1% from the nominal values to simulate realistic elastic strain states. Synthetic diffraction data were generated using the forward simulation module of *MIDAS*, with an omega step of 0.25° over 360° and a simulated spot width of 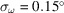 in the rotation direction, producing realistic multi-frame diffraction peaks. To assess robustness under realistic conditions, the experiment was performed both with perfect data and with Gaussian spot position noise of 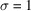 pixel applied to each diffraction peak. The complete FF-HEDM pipeline (peak search, indexing, refinement, consolidation) was then executed on each synthetic dataset.

*Comparison methodology*. Two refinement strategies were compared using the same indexed spots and identical total computational budget (40000 Nelder–Mead function evaluations):

(*a*) Iterative (*MIDAS*). The four-stage decoupled scheme described in Part I: (1) 12-parameter position fit using 2D detector position error, (2) 9-parameter orientation fit using 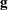 angular matching, (3) 6-parameter strain fit using 2D position error with fixed position and orientation, and (4) 3-parameter position refinement.

(*b*) All-at-once. Simultaneous optimization of all 12 parameters (3 position + 3 orientation + 6 lattice) using a single 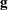 angular mismatch objective, representative of approaches commonly used in the literature (*e.g.* Oddershede *et al.*, 2010 ▸; Bernier *et al.*, 2011 ▸).

Grains were matched between the two reconstructions and the known ground truth using the Hungarian algorithm with symmetry-aware misorientation (space group 225) via the grain matching framework described in Part I. For lattice parameter accuracy in cubic crystals, the three lattice parameters *a*, *b* and *c* are symmetrically equivalent, so the optimizer may converge to any permutation of their values. To ensure a fair comparison, we evaluate lattice parameter accuracy using a permutation-aware metric: for each grain, we compute the error for all six permutations of the fitted lattice parameter 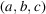 against the ground truth and report the minimum.

*Per-stage convergence*. To make the contribution of each stage of the decoupled refinement explicit, we logged the residual errors after each of the four stages. Table 8 ▸ shows the mean residuals across all 250 grains for the noisy (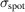 = 1 pixel) case. Each successive stage progressively reduces the residual: stage 1 (12-parameter position fit) brings the position error to 215 µm; stage 2 (orientation refinement) further reduces position error to 184 µm and halves the omega and angular errors; stage 3 (dedicated strain fit) provides the largest single improvement, reducing the position residual by ∼30% to 131 µm; stage 4 provides marginal final position refinement. The progressive improvement confirms that each stage contributes meaningfully and that the parameter groups benefit from being optimized independently.

**Figure 9:**
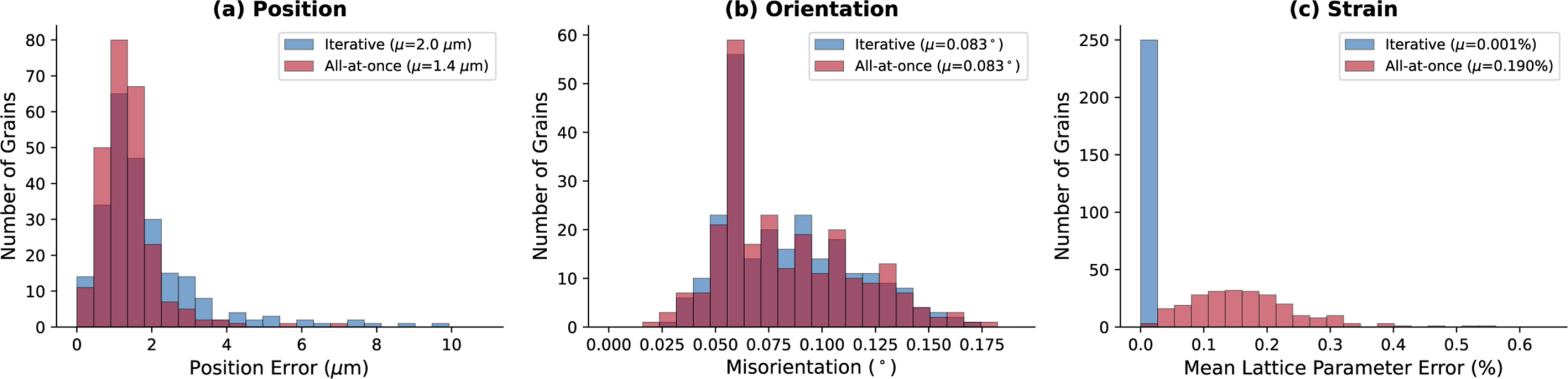
FF_HEDM: comparison of iterative (blue) versus all-at-once (red) refinement using 250 simulated f.c.c. grains with known ground truth. (*a*) Position error distribution. (*b*) Orientation error (misorientation) distribution. (*c*) Mean lattice parameter error distribution, computed using a permutation-aware metric that accounts for the cubic symmetry equivalence of *a*, *b* and *c*. Both methods recover position and orientation with comparable accuracy, but the iterative approach achieves 190× better lattice parameter precision (μ = 0.001% versus 0.190%).

*Results*. All 250 grains were successfully indexed and refined by both methods in both noise conditions. Fig. 9 ▸ shows the distributions of errors for the noise-free case. Position accuracy and orientation accuracy are comparable between the two methods, with mean errors of ∼2 µm and ∼0.083°, respectively. This is expected because the 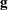 angular objective used by the all-at-once method directly targets orientation and position through the geometric relationship between observed and theoretical diffraction vectors.

The critical difference emerges in lattice parameter precision. Table 9 ▸ summarizes the results for both noise conditions. With perfect synthetic data, the iterative approach achieves a mean lattice parameter error of 0.001% compared with 0.190% for the all-at-once method, a factor of 190× improvement. Under realistic noise (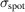 = 1 pixel), the iterative method achieves 0.011% versus 0.252% for the all-at-once approach, a factor of 24× improvement. The advantage of the iterative scheme persists and remains substantial under realistic measurement noise.

This dramatic improvement arises because the iterative scheme’s dedicated strain-fitting stage (stage 3 in Part I, Section 3.5) operates in a reduced 6D parameter space with position and orientation fixed, using a 2D detector position objective that is directly sensitive to *d*-spacing changes through the radial position of diffraction peaks. In contrast, the all-at-once approach must simultaneously navigate a 12D landscape where orientation and strain parameters are tightly coupled, and the angular objective has inherently lower sensitivity to small lattice parameter perturbations. The 0.001% residual error of the iterative method (no noise) corresponds to ∼2 µm (0.01 pixels) on the detector at the highest ring used, confirming that the refinement is recovering the ground truth lattice parameters to within sub-pixel precision.

Notably, the iterative scheme is also *computationally faster* than the all-at-once approach (23 s versus 35 s for the refinement of all 250 grains using eight CPU cores), despite both methods using the identical total budget of 40000 Nelder–Mead function evaluations. This is because the lower-dimensional sub-problems in stages 2–4 (9, 6 and 3 parameters, respectively) converge in fewer iterations than the full 12D optimization, even though the maximum allowed budget is the same. The iterative scheme thus delivers both higher accuracy and lower runtime, contradicting the intuitive expectation that decoupling parameters would require more computational effort.

## 3. NF-HEDM

### 3.1. Experimental details

Concurrently with the FF-HEDM experiments, a set of NF-HEDM datasets were acquired on the same sample regions. The same X-ray beam (*E* = 65.351 keV, 0.9 µm × 2.0 mm H × V) was incident on the sample and diffraction data were acquired for multiple sample-to-detector distances, from ∼4.5 mm to ∼8.5 mm in 1.0 mm increments. The detector position was optimized such that the direct beam would be incident towards the vertical bottom edge of the detector, and a tungsten beam block, placed just before the detector, was used to attenuate the direct beam signal on the detector. For each detector distance, diffraction images were acquired with a rotation step size of 0.25° over a total range of 180°. Such datasets were acquired multiple times at multiple heights in the sample, and nine datasets were collected, described in Table 10 ▸. In each case, the sample was either intentionally moved in the *z* (vertical) direction, or the starting angle for the rotation was changed, or both. An additional dataset with a total rotation range of 360° was also collected.

### 3.2. Reconstruction details

An initial guess of the experimental setup was obtained using an Au-cube sample with 3–4 grains centered around the rotation axis. The experimental parameters were then refined using the sample data and are given in Table 11 ▸. Leveraging the synergy between FF- and NF-HEDM within the framework (as discussed in Part I, Section 4.4), the candidate orientation list for the NF-HEDM reconstructions was initially seeded using the grain orientations determined from the FF-HEDM analysis (Section 2.3.1). This significantly reduces the computational search space compared with a full brute-force approach. Analysis was run on an HPC with five nodes, each with 128 AMD EPYC CPU cores. Similar to FF-HEDM, NF-HEDM was computationally efficient, taking about 3 min to finish per dataset. The raw diffraction data were first pre-processed using the removal of a background computed as a through-data median filter. A Laplacian-of-Gaussian filter was then applied to determine peak edges, followed by a threshold filter of ten counts on the remaining non-zero intensity pixels. This dataset was then binarized: each non-zero intensity pixel was set to one, and all the other pixels were set to zero.

A triangular grid with equilateral triangles with edge length of 2 µm was used to generate the reconstruction space in a box of 1200 × 200 µm, centered around the rotation axis (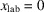 and 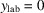). The µ-CT reconstruction was used to filter out the sample reconstruction space, with only voxels inside the sample used for NF-HEDM reconstruction. Applying such a mask is crucial for samples with non-trivial geometry to prevent reconstruction artifacts and wasted computation outside the material volume; the framework’s ability to incorporate this information (Part I, Section 4.3) is key for practical analyses.

The candidate orientations list was generated using the results from the reference FF-HEDM reconstruction described in Section 2.3.1. Furthermore, during the reconstruction, all the voxels with Confidence < 0.75 were searched again using a candidate orientation list comprising uniformly sampled orientations in the fundamental zone for hexagonal close-packed (h.c.p.) crystal structures with a spacing of ∼2°. A deviation of up to 4° in the orientation of each voxel from each candidate orientation (FF-HEDM seed or uniformly sampled) was used during orientation refinement. This refinement step (Part I, Section 4.5) is vital for correcting small inaccuracies in the initial seed orientations and achieving the high angular accuracy demonstrated below.

### 3.3. Results and discussion

#### 3.3.1. Reference reconstruction

**Figure 10:**
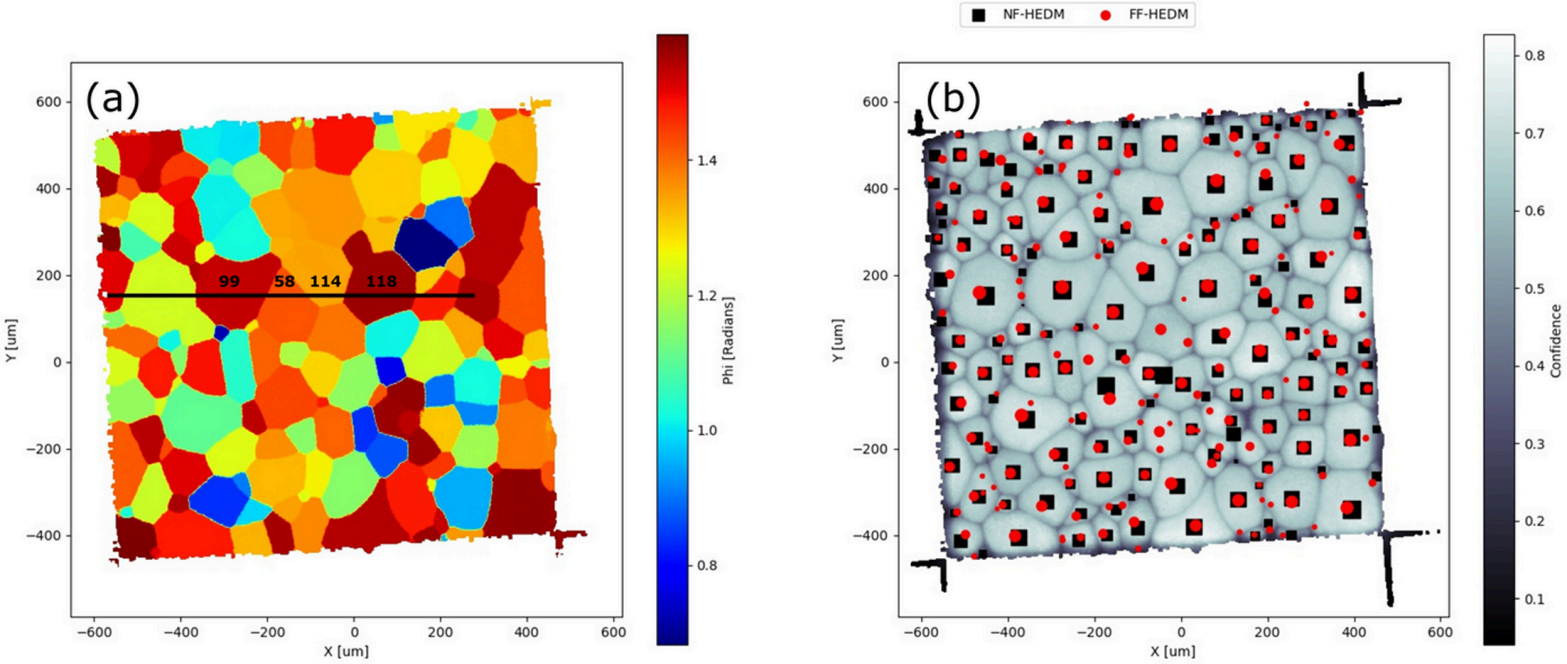
Reference NF-HEDM reconstruction. (*a*) Colored by the Euler angle (ϕ, radians). (*b*) The position of the COM of grains computed using NF-HEDM (black squares) and FF-HEDM (red circles) overlaid on top of the NF-HEDM confidence map. The size of the FF-HEDM markers is proportional to the computed grain size.

**Figure 11:**
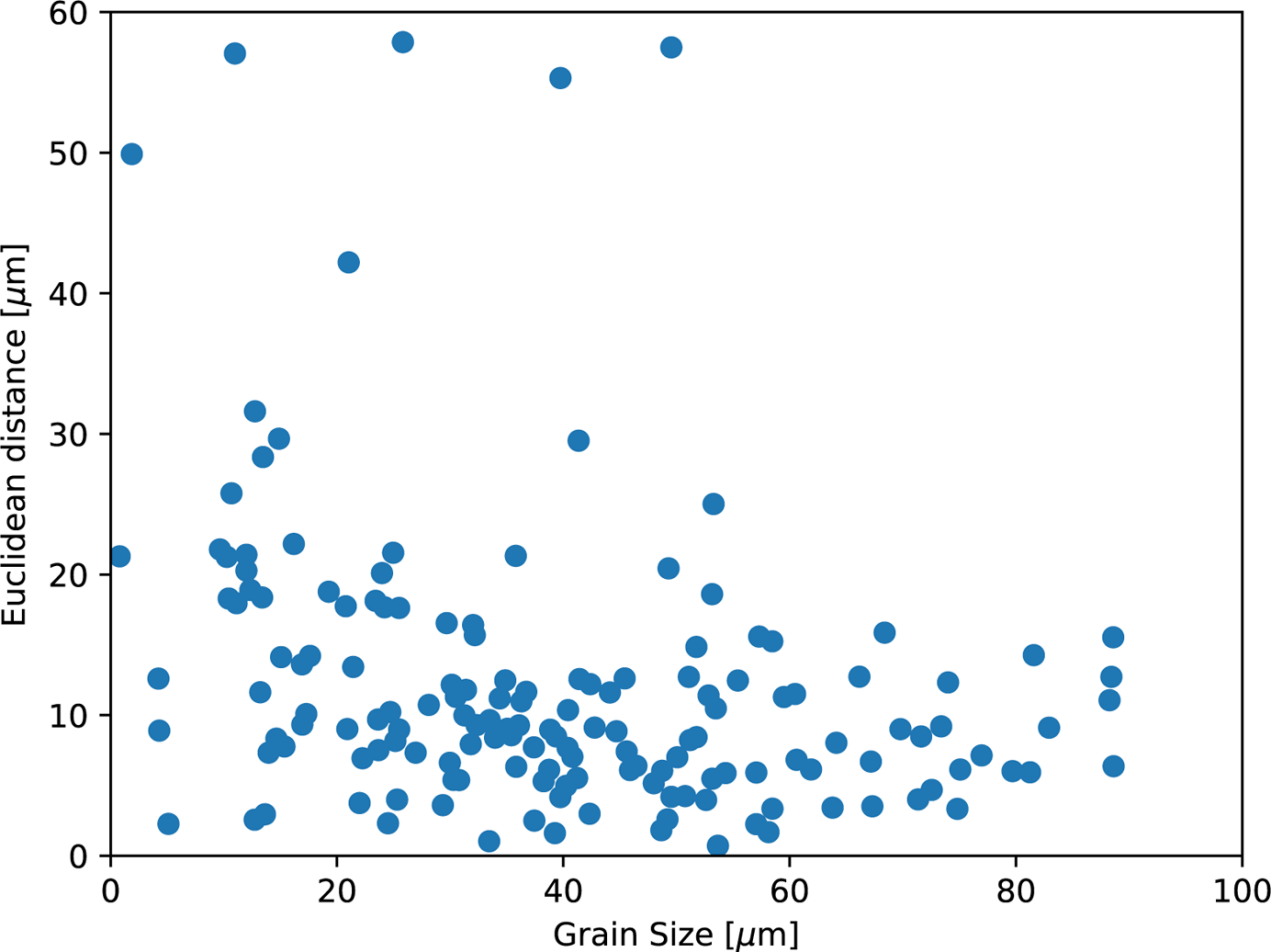
Euclidean distance between the COM of grains computed using NF-HEDM and FF-HEDM as a function of grain size computed from NF-HEDM.

The reference reconstruction for dataset 0 in Table 10 ▸ is shown in Fig. 10 ▸. The edge artifacts outside the sample are reconstruction artifacts from the µ-CT reconstruction. Fig. 10 ▸(*b*) shows the center-of-mass (COM) position of the grains computed using NF-HEDM (black squares) and FF-HEDM (red circles, computed from the reference reconstruction described in Section 2.3.1). The COM position of grains in NF-HEDM was calculated by using a misorientation threshold of 5°; that is, during grain search, a new grain is only detected if the smallest misorientation with all existing grains is greater than 5°. It can be seen that most of the COMs from NF-HEDM and FF-HEDM are very close [mean Euclidean distance 12.94 µm (Fig. 11 ▸)], validating the general consistency between the two methods as implemented in the framework. This successful use of FF-HEDM orientations to seed the NF-HEDM reconstruction demonstrates a powerful synergy that dramatically reduces computation cost compared with a brute-force orientation search. The trend in Fig. 11 ▸, where larger grains tend to show smaller COM differences, likely reflects better signal statistics in both techniques for larger volumes and less influence from boundary voxel assignments in the NF-HEDM COM calculation. Smaller grains exhibit larger discrepancies, possibly due to weaker signals approaching detection limits (especially the 12-bit NF detector) and the NF-HEDM COM being more sensitive to voxelation and boundary segmentation choices.

While the 12-bit dynamic range of the NF-HEDM detector (compared with 14-bit for FF-HEDM) limits the overall signal ceiling, it is important to note the difference in intensity scaling. FF-HEDM peak intensities scale with the total grain volume, whereas NF-HEDM intensities scale with the grain volume projected onto a given pixel. Consequently, small but well ordered grains may yield sufficient per-pixel intensity to be detected in NF-HEDM even if missed in FF-HEDM, meaning the missing grains are likely a complex interplay of misorientation thresholds, bit depth and these projection scaling effects.

The missing grains (26) from NF-HEDM can be attributed to:

(i) Grains with misorientation less than 5° that could be resolved using FF-HEDM but were not resolved using the larger misorientation threshold used for NF-HEDM grain segmentation.

(ii) Small grains, so that the diffraction signal was not observed in NF-HEDM due to the limited dynamic range of the detector: 12-bit versus 14-bit for NF-HEDM and FF-HEDM, respectively. This highlights the inherent sensitivity limit.

**Figure 12:**
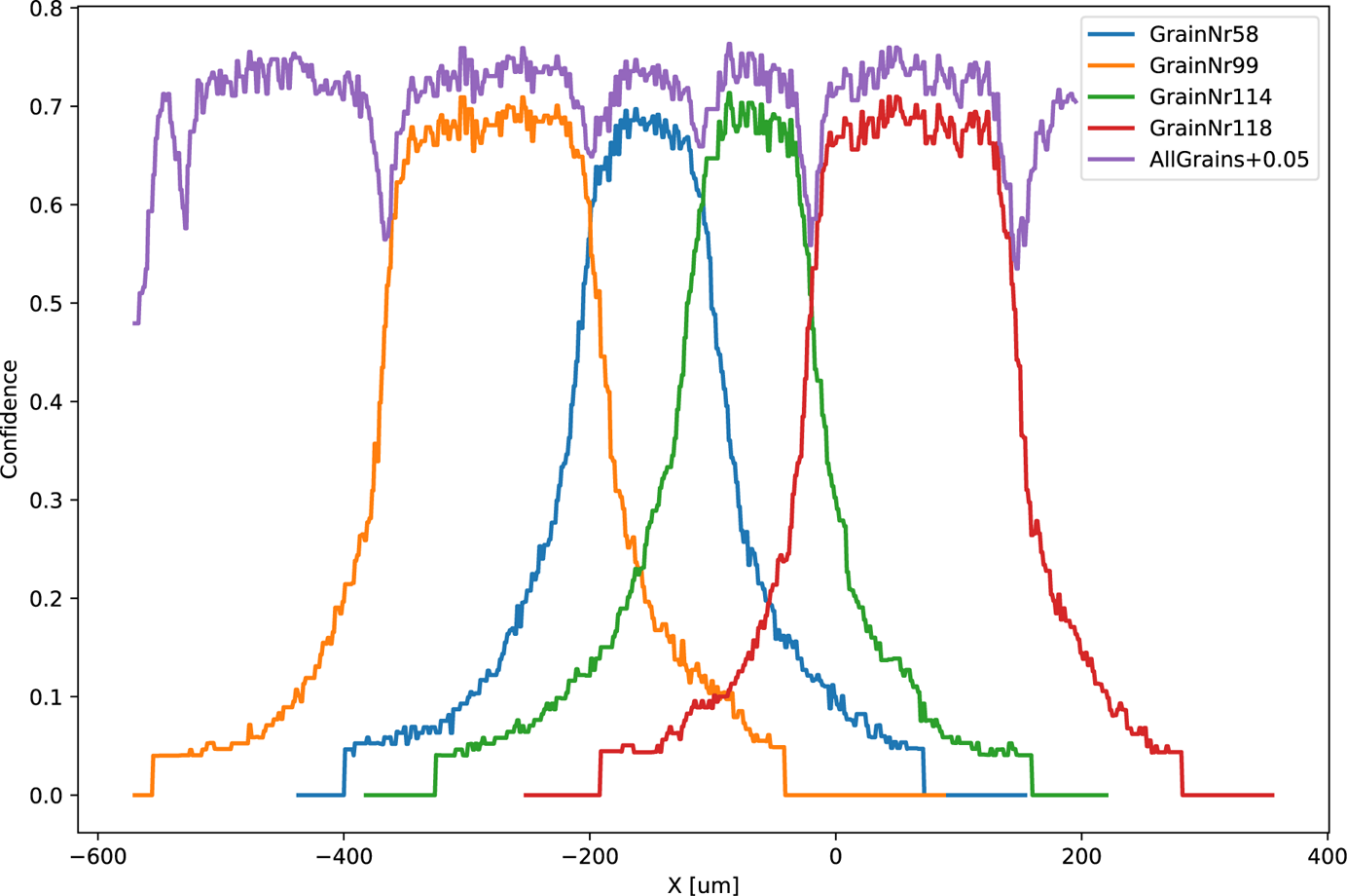
NF_HEDM: evolution of confidence along the black horizontal line in Fig. 10 ▸(*a*) for the different grains (blue, orange, green and red lines) and overall (purple line). The overall confidence (purple line) is vertically displaced by 0.05 for illustrative purposes.

Fig. 12 ▸ shows the evolution of confidence for different orientations along a line through several grains. The overall confidence (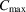) peaks within each grain and transitions sharply at grain boundaries. This illustrates the principle of NF-HEDM reconstruction within the framework: the orientation yielding the maximum confidence at a voxel determines its assignment. Grain boundaries are thus implicitly defined at locations where the maximum confidence (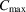) switches from one orientation solution to another; voxels near the boundary may show moderate confidence values for multiple orientations before one clearly dominates. It also highlights a nuance: NF-HEDM primarily defines internal grain boundaries where orientation changes dominate; external sample edges are typically defined using complementary data like µ-CT, as done here.

#### 3.3.2. Parameter study

To evaluate the sensitivity of the framework’s NF-HEDM reconstruction to experimental choices and guide optimal data acquisition strategies, different experimental configurations were simulated or analyzed.

**Figure 13:**
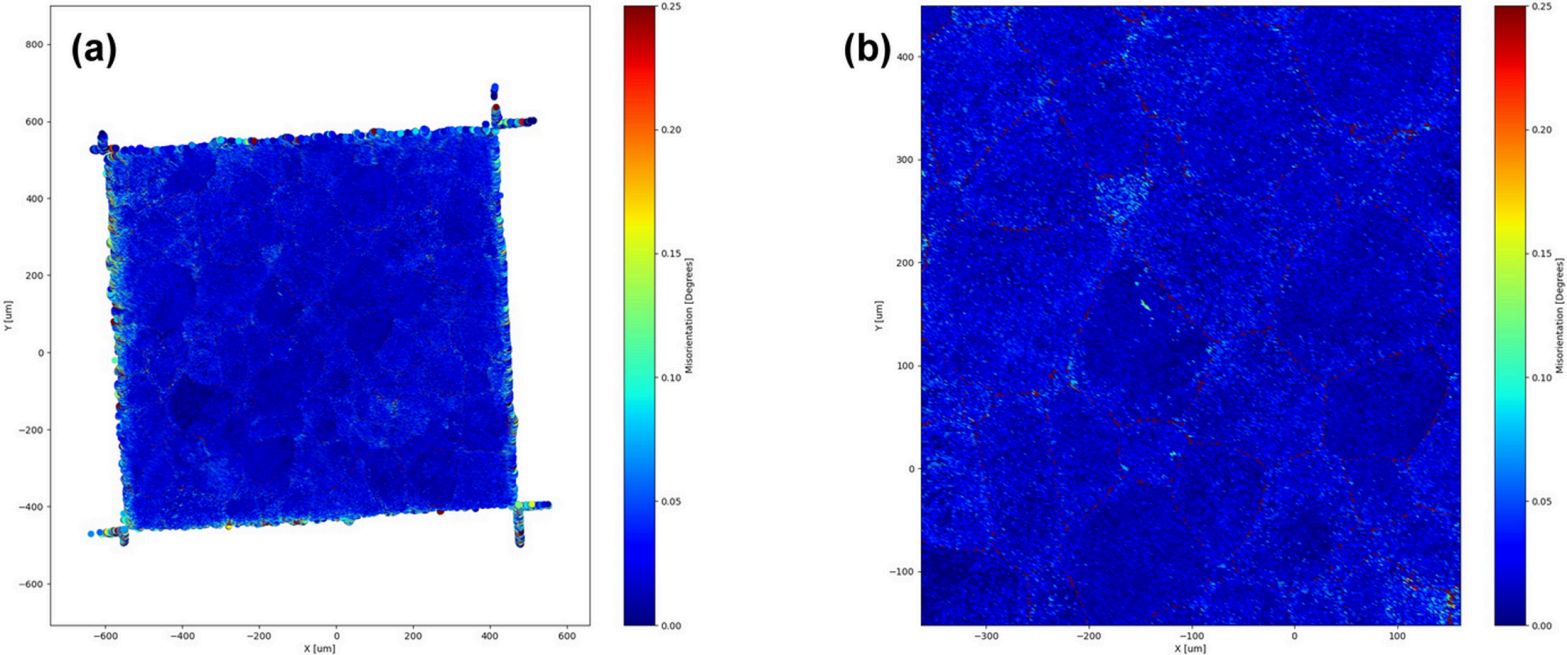
NF_HEDM: plot of misorientation angles between the reference and D1D5 reconstructions. (*a*) Full reconstruction. (*b*) Zoomed-in view. For more details, readers are directed to the text.

**Figure 14:**
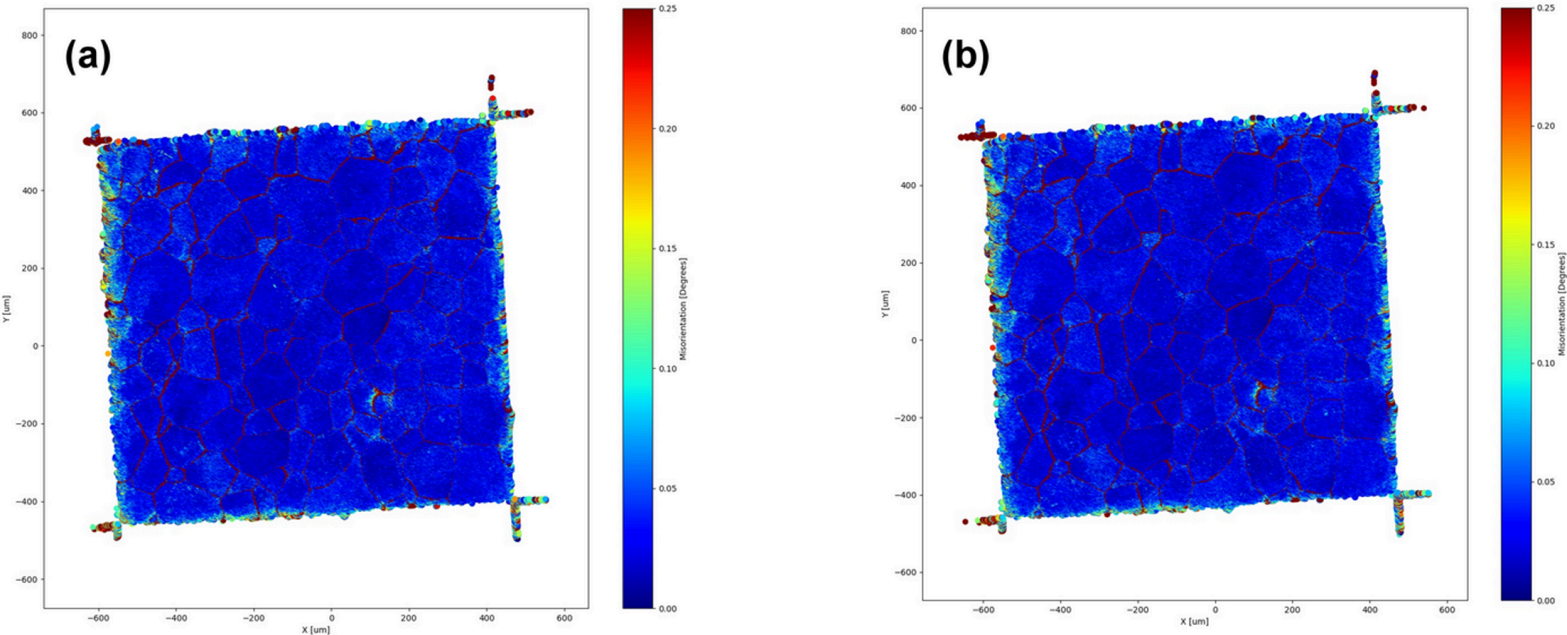
NF_HEDM: plot of misorientation angles between (*a*) D1D2 and reference reconstructions, and (*b*) D1D2 and D1D5 reconstructions. For more details, readers are directed to the text.

*The impact of triangulation baseline on NF orientational precision*. Reconstructions were performed using only the nearest and farthest detector positions (D1D5, Fig. 13 ▸) and only the two nearest positions [D1D2, Fig. 14 ▸(*a*)]. Comparing these with the reference (using all five distances) and each other [Fig. 14 ▸(*b*)] reveals a critical finding: using detector positions farther apart (D1D5) yields significantly higher accuracy (mean misorientation 0.022° versus reference) compared with using closer positions (D1D2, mean misorientation 0.028° versus reference, with more boundary discrepancies). This confirms the theoretical nuance: larger triangulation baselines improve reconstruction precision. A key strength of the framework is its ability to achieve high accuracy using only two detector distances, significantly saving acquisition time compared with using all five. Best practice: maximize the distance between the two (or more) detector positions used for NF-HEDM acquisition within experimental constraints (*e.g.* detector size, beamstop position). However, researchers must balance this baseline maximization. Using two detectors with excessively large separation will eventually reduce the number of high-angle diffraction peaks that strike the far detector, which can reduce overall orientational precision. Furthermore, very large distances can increase sensitivity to the divergence of the diffracted beam caused by beam energy bandwidth and the use of focusing optics.

**Figure 15:**
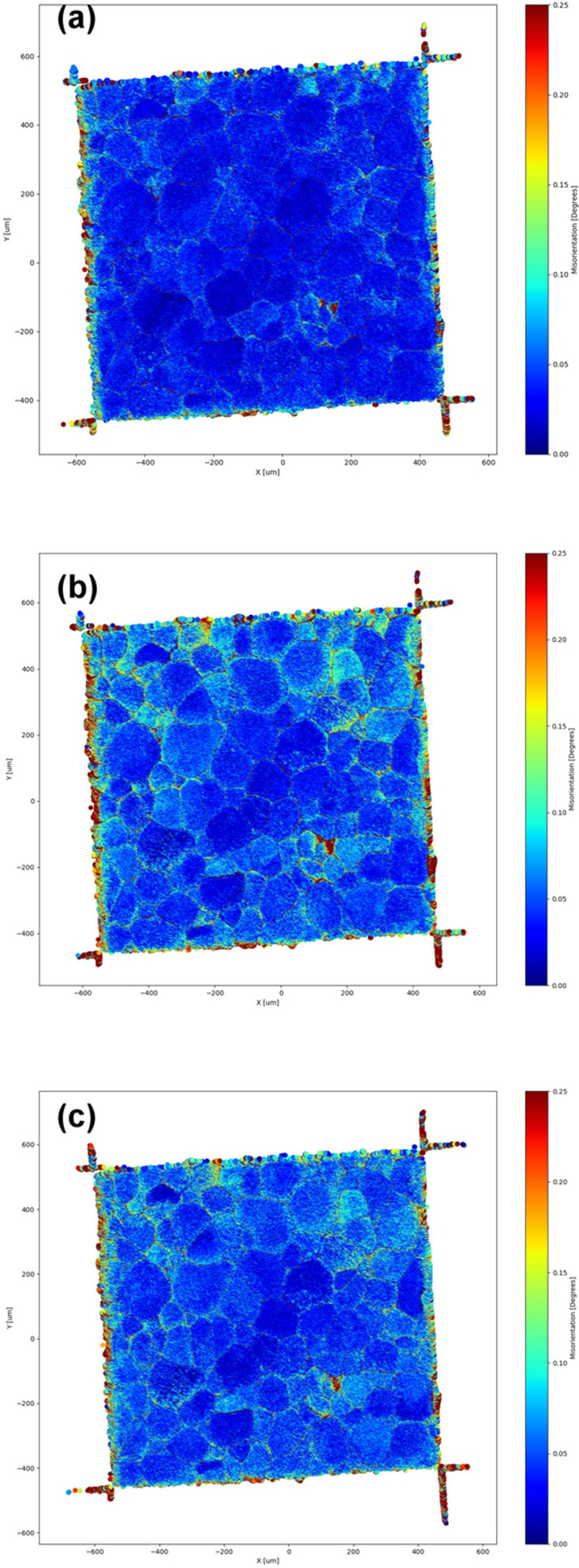
NF_HEDM: plot of misorientation angles between (*a*) 0.5° and reference reconstructions, (*b*) 1° and reference reconstructions, and (*c*) 0.5° and 1° reconstructions. The 0.5° and 1° reconstructions used generated data by summing every two and four diffraction images. For more details, readers are directed to the text.

*Dependence of reconstruction fidelity on angular sampling*. Virtual datasets with 0.5° and 1° rotation steps were created by summing adjacent frames from the 0.25° reference dataset. Reconstructions using these coarser steps showed at least 73% higher mean misorientation compared with the reference [mean 0.038° for 0.5°, 0.058° for 1°, Figs. 15 ▸(*a*), 15 ▸(*b*) versus 0.022° for reference], particularly at grain boundaries. This highlights the nuance that finer angular sampling improves reconstruction fidelity. The comparison between the 0.5° and 1° reconstructions [mean misorientation 0.055°, Fig. 15 ▸(*c*)] shows similar grain boundary locations but different internal misorientations compared with changing detector distances [Fig. 14 ▸(*b*)]. Best practice: use the smallest practical rotation step size, ideally matching the detector’s angular resolution and the scale of expected microstructural features.

**Figure 16:**
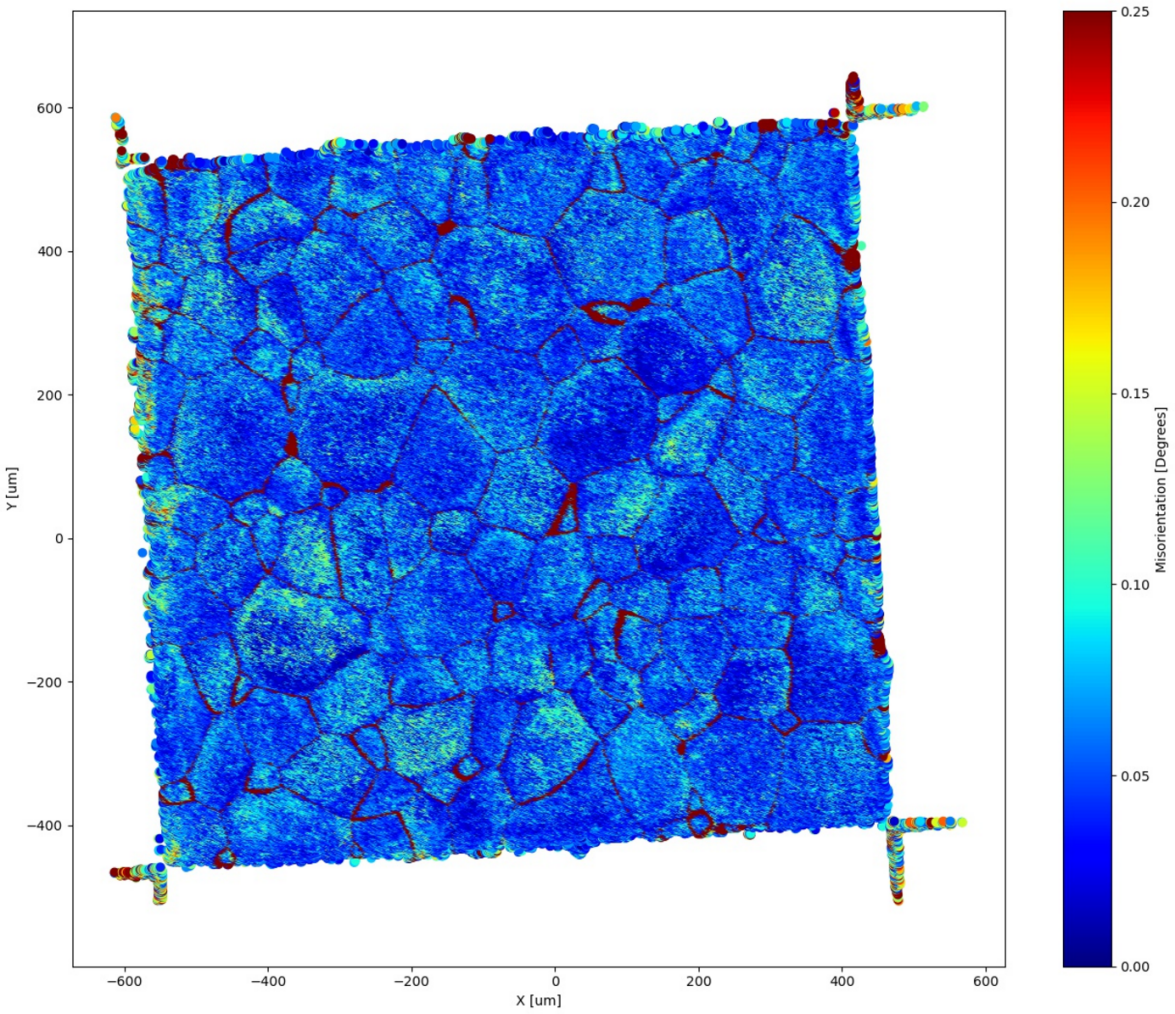
NF_HEDM: plot of misorientation angles between datasets 7 and 9 from Table 10 ▸. The two datasets were acquired on two regions of the sample vertically apart by 4 µm.

**Figure 17:**
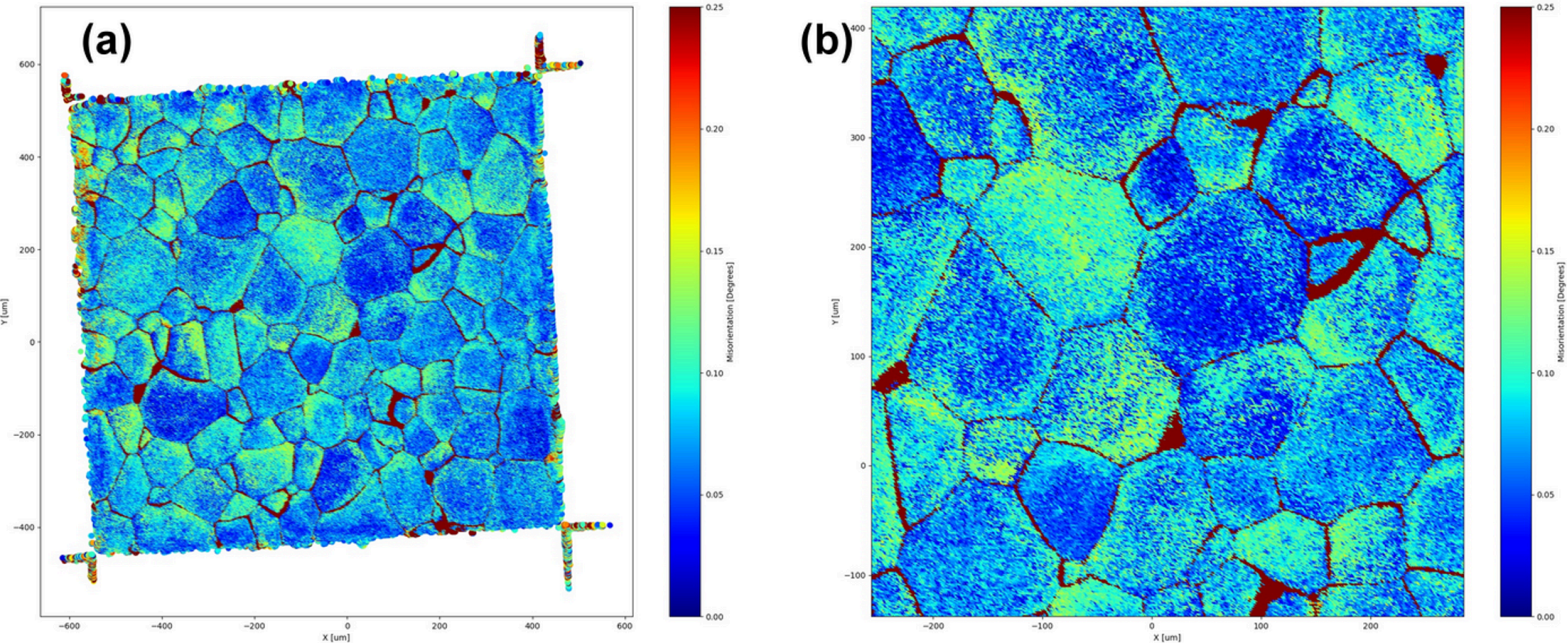
NF_HEDM: plot of misorientation angles between datasets 6 and 9 from Table 10 ▸. The two datasets were acquired on the same region of the sample but with a different starting rotation angle, apart by 0.1°. (*a*) Full reconstruction. (*b*) Zoomed-in view highlighting the variation in misorientation angles between different grains.

*Robustness of seeded reconstructions to geometric mis­alignment*. Datasets 1–9 involved known vertical shifts (*z*) or starting angle shifts (ω) relative to the FF-HEDM data used for seeding orientations (Table 10 ▸). Reconstructions were performed using the mismatched FF-HEDM seeds. The resulting mean misorientations (Table 12 ▸) closely tracked the known ω displacement, with standard deviations consistently low (<0.15× rotation step) and the difference between known displacement and mean misorientation below 0.018°. Comparing reconstructions from layers 4 µm apart (datasets 7 and 9, Fig. 16 ▸) shows misorientations <0.1° within grain interiors, confirming robustness. This demonstrates a crucial strength of the framework: its robustness to small, realistic experimental variations between scans or between the FF-HEDM seed data and the NF-HEDM target data. This is vital for reliable layer-by-layer 3D reconstructions. Even non-uniform misorientation distributions relative to the applied ω shift (Fig. 17 ▸) were handled, indicating robustness to minor microstructural variations or seed inaccuracies.

**Figure 18:**
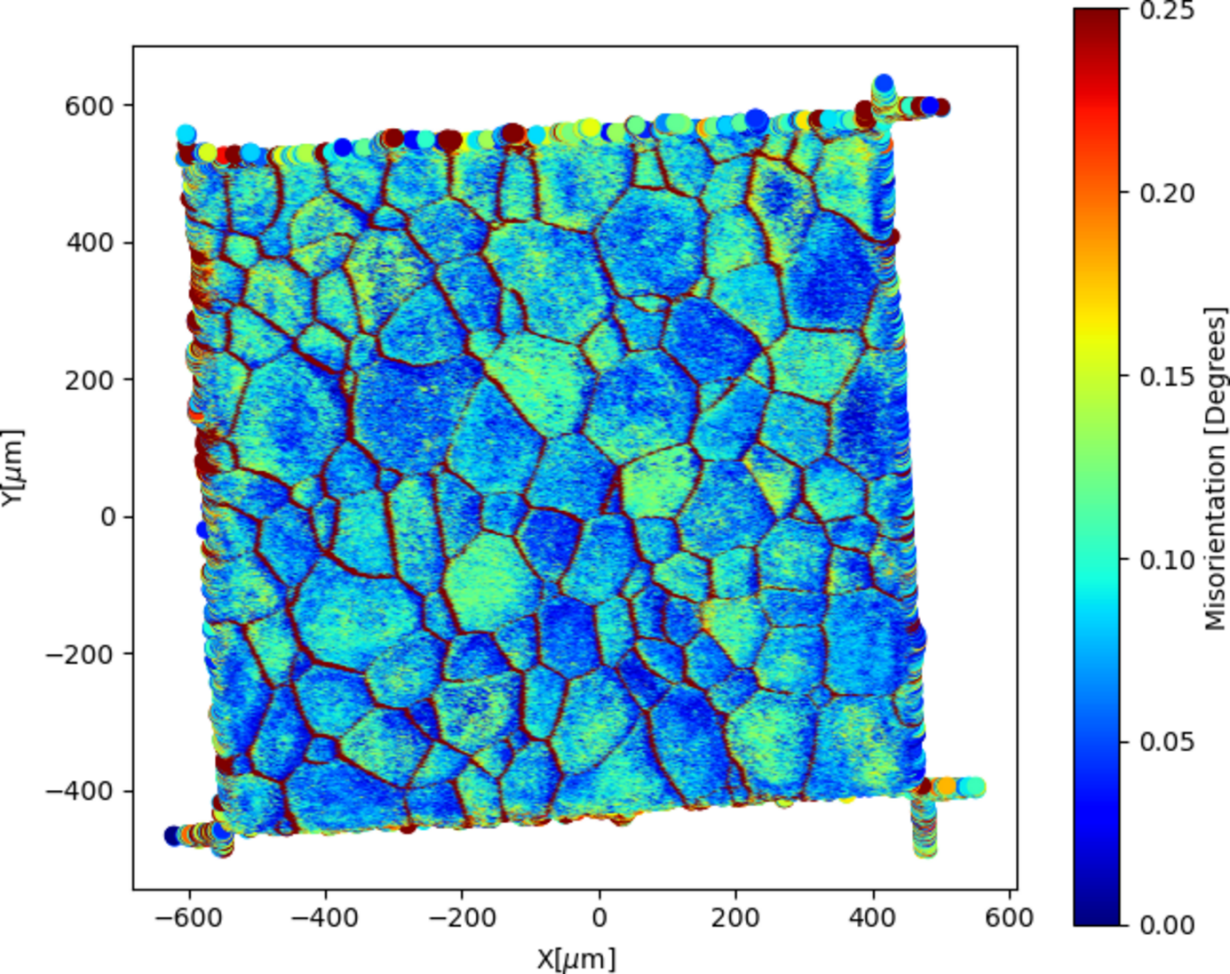
NF_HEDM: plot of misorientation angles between the reference reconstruction and reconstruction of dataset 10 from Table 10 ▸. The two datasets were acquired on the same sample region but with a different rotation range (180° and 360° for datasets 0 and 10, respectively).

*Impact of rotation range*. Comparing the reference 180° reconstruction (dataset 0) with a 360° reconstruction (dataset 10, Fig. 18 ▸) showed a slightly higher mean misorientation (0.077° versus ∼0.02–0.06° in other comparisons). While differences are concentrated near grain boundaries, this suggests a subtle nuance: 360° rotation might capture slightly more information or lead to different boundary definitions, although the overall increase in mean misorientation warrants further investigation. Best practice: for most applications, 180° rotation offers a significant time saving and provides high-quality reconstructions, as validated here. Rotation of 360° might be considered only if highest precision at boundaries is paramount and the extra time is justified.

Overall NF-HEDM validation: the results validate the framework’s NF-HEDM pipeline, demonstrating accurate reconstructions that leverage FF-HEDM data for efficiency. Key recommendations for optimal data acquisition are maximizing detector separation and using fine rotation steps. The software shows significant robustness to typical experimental variations, making it a practical tool for time-series and 3D mapping experiments.

### 3.4. Probing the framework’s limitations and future directions

To explore the nuances of NF-HEDM data processing further and identify areas for potential improvement within the framework, a publically available dataset from Sparks *et al.* (2024 ▸) was utilized. This dataset is unique because it consists of FF-HEDM, NF-HEDM, EBSD and surface profilometry data from the same regions of the sample. These complementary techniques can be used to compare and evaluate the performance of the techniques and data analysis algorithms. Building upon the published results, we investigate the limitations of current data analysis techniques and discuss ways to improve the analysis.

**Figure 19:**
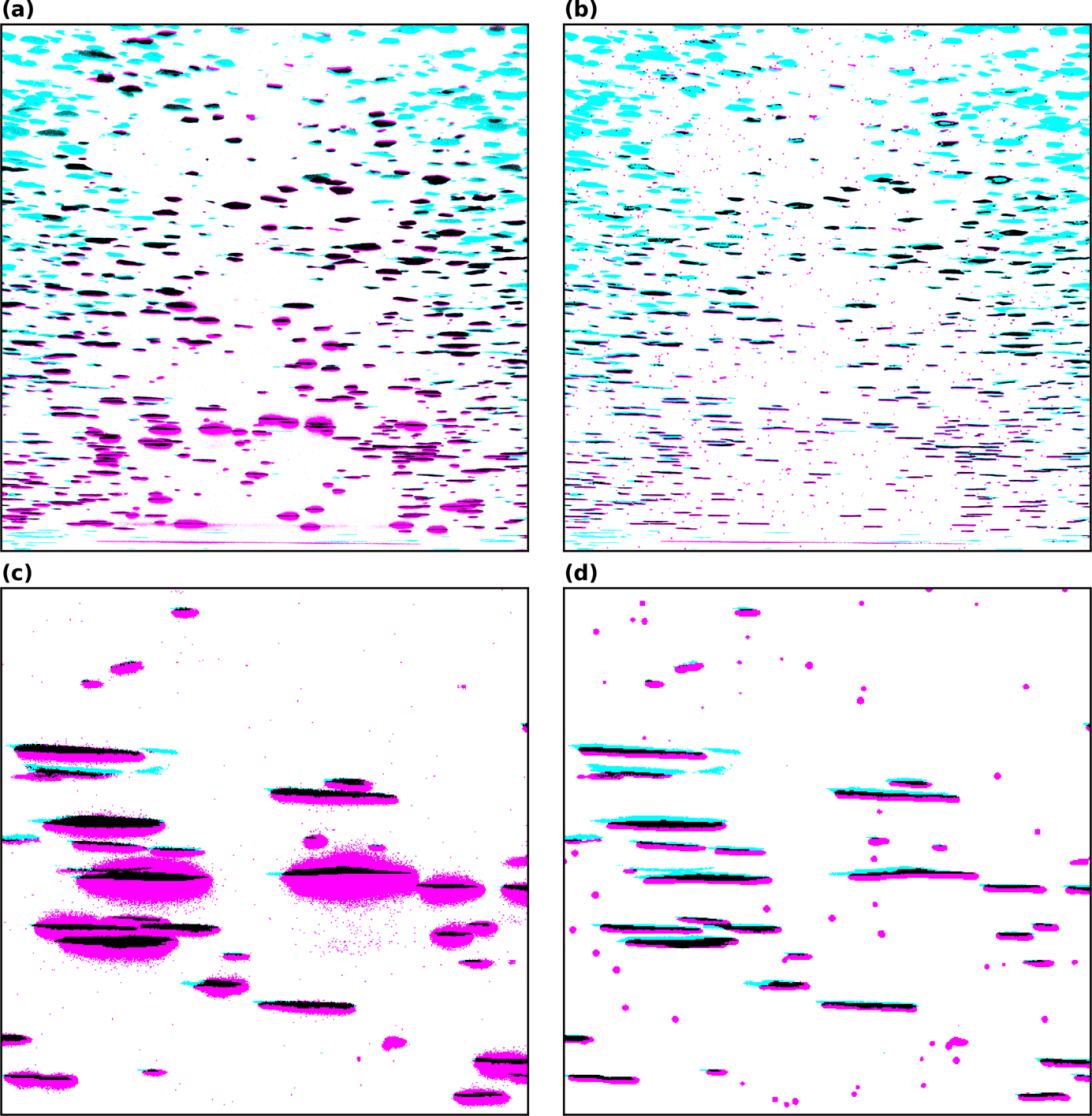
NF_HEDM: plot of diffraction data and simulated data from the microstructure for different image processing techniques. The color scheme is: cyan for pixels with simulated signal only, magenta for pixels with observed signal only, and black for pixels with both simulated and observed signal. (*a*) Cleaned diffraction image without Laplacian-of-Gaussian (LoG) filter. (*b*) Cleaned diffraction image with LoG filter. (*c*) Zoomed-in view of (*a*). (*d*) Zoomed-in view of (*b*).

As reported by Sparks *et al.* (2024 ▸), some grains detected in EBSD were missed in the original NF-HEDM analysis. Using the EBSD map as ground truth, we simulated the expected NF-HEDM signal (summing 30 frames centered at 0° for adequate signal). Fig. 19 ▸ compares this simulation with the experimental NF-HEDM data, both before (*a*, *c*) and after (*b*, *d*) applying the standard Laplacian-of-Gaussian (LoG) filter typically used for edge enhancement in peak detection (Part I, Section 4.2).

Several important observations and analysis nuances arise:

(i) Higher-order peaks (top of detector). Simulated signal (green) is often present where experimental signal (blue/red) is absent. This top region corresponds to the high-*Q* region of reciprocal space. The absence of experimental signal here is physically consistent with the atomic form factor, which inherently reduces scattering intensities at higher diffraction angles, frequently pushing these weak signals below the detector’s background noise or detection limits.

(ii) Lower-order peaks (bottom of detector). Before LoG filtering [Figs. 19 ▸(*a*), 19 ▸(*c*)], experimental peaks (blue/red) are often vertically larger than simulated peaks (green/red). Applying the LoG filter [Figs. 19 ▸(*b*), 19 ▸(*d*)] improves the match for these lower-order peaks but degrades the match for the (already weak) higher-order peaks.

(iii) Shape information. The vertical extent of experimental peaks [red pixels in Fig. 19 ▸(*c*)] is larger for higher-order peaks, consistent with projection effects being more pronounced at higher diffraction angles, potentially offering better grain shape information.

These observations highlight a challenge in NF-HEDM processing: applying a single image processing strategy (like the LoG filter) across the entire detector may sub-optimally extract information, potentially improving edge detection for strong low-angle peaks while suppressing weaker high-angle peaks. Capturing maximum information from higher-order peaks is desirable due to better shape sensitivity.

This challenge also points to a potential strength and future direction for the framework. Its current NF-HEDM pipeline relies on binarized images after uniform processing. However, the framework’s modular design (Part I) separates image processing from the core reconstruction algorithms that work with peak/voxel information. This architecture offers a pathway to address the filtering dilemma: advanced workflows could involve applying different processing steps (*e.g.* LoG filter, thresholding, no filter) to different detector regions or *hkl* families before peak detection or segmentation. The resulting tailored lists of detected pixels/peaks from different regions could then be combined and fed into the existing reconstruction engine. Such an approach, enabled by the framework’s structure, could potentially optimize signal recovery across the full range of diffraction angles, improving both completeness and shape reconstruction in NF-HEDM. Addressing this processing challenge could further enhance the accuracy and utility of NF-HEDM, particularly for studies requiring precise grain shape information derived from higher-order reflections.

## 4. Experimentally derived operational principles for HEDM analysis

Based on the systematic validation studies presented in this work, we propose the following evidence-based best practices for researchers conducting HEDM experiments and analysis. These recommendations are designed to maximize data quality, accuracy and reproducibility.

(i) For FF-HEDM analysis:

(*a*) To maximize completeness. The RingToIndex parameter, which selects the diffraction ring for generating initial grain orientation candidates, should be set to the most intense, complete ring available. Our results show this significantly increases the probability of identifying smaller grains that may only diffract weakly on other rings (see Section 2.3.2).

(*b*) To maximize accuracy. The RingsToAnalyze parameter should include as many reliable diffraction rings as practical, especially those at higher 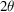 angles, to provide superior geometric constraints for position and strain refinement.

(ii) For NF-HEDM acquisition and analysis:

(*a*) To maximize orientation precision. The physical separation between the first and last detector positions should be maximized within the constraints of the experimental setup. Our validation demonstrates a direct correlation between this triangulation baseline and the resulting orientation accuracy, with larger separations yielding dramatically lower mis­orientation errors (see Section 3.3.2).

(*b*) To define sample boundaries. For non-trivial sample geometries, it is essential to use a complementary dataset (*e.g.* µ-CT) to create a mask for the reconstruction volume. This prevents wasted computation and avoids reconstruction artifacts at the sample edges.

(iii) For general and integrated analysis:

(*a*) Leverage FF–NF synergy. Whenever possible, use results from a companion FF-HEDM scan to seed the candidate orientations for NF-HEDM reconstruction. This massively reduces computational cost and has been shown to be robust to minor experimental misalignments.

## 5. Summary

This paper has established a quantitative and systematic validation paradigm for complex, large-scale diffraction methodologies. By investigating the HEDM data reduction framework presented in Part I, we have moved beyond simple software validation to define its fundamental operational principles and performance limits. Our findings provide a direct, quantitative link between experimental and analysis parameters and the final accuracy of microstructural reconstructions. The demonstrated importance of 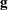 diversity in far-field analysis and triangulation baseline in near-field analysis provide clear, actionable principles for the design of robust experiments. Finally, we have synthesized these findings into a framework of best practices that will enable the broader scientific community to conduct more accurate, reliable and reproducible HEDM studies. While the exact numerical error bounds and parameter sensitivities detailed in this study are inherently tied to the specific Ti-7 Al microstructure (*e.g.* its grain size distribution and mosaicity), the underlying trends – such as the vital role of 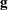 diversity in far-field accuracy and triangulation baseline in near-field precision – represent universal methodological principles applicable across crystalline materials.

## Figures and Tables

**Table 1:** Refined parameters of the experimental setup for the FF-HEDM experiment

*L* (µm)	940710.1499
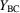 (pixels)	1024.7291
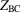 (pixels)	994.3471
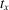 (°)	−0.0804
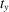 (°)	−0.2614
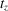 (°)	0.0107
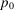	4.9819 × 10^−5^
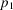	−6.8098 × 10^−5^
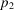	−4.8345 × 10^−5^
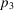 (°)	20.3788

**Table 2:** Reference crystal parameters for the Ti 7 Al sample

*a*	*b*	*c*	α	β	γ	Space group
2.92658586 Å	2.92658586 Å	4.67473 Å	90°	90°	120°	194

**Table 3:** Diffraction rings for the Ti-7 Al sample For the column ‘theoretical grains’ 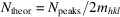.

Ring No.	*hkl*	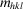	Ring radius (µm)	Attenuated	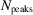	Theoretical grains	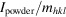 (arbitrary units)
1	100	6	70563.023	Yes†	N/A	N/A	N/A
2	002	2	76542.655	Yes†	N/A	N/A	N/A
3	011	12	80316.514	Yes†	N/A	N/A	N/A
4	012	12	104343.266	No	4674	195	1716
5	110	6	122736.802	No	3214	268	5748
6	013	12	135484.690	No	4994	208	2462
7	012	6	142025.791	No	2244	187	1196
8	012	12	**145167**.**454**	No	N/A	N/A	N/A
9	012	12	**147243**.**972**	No	N/A	N/A	N/A
10	012	2	154235.188	No	852	213	2126
11	012	12	161962.649	No	3889	162	601
12	012	12	170205.873	No	3654	152	374
13	012	12	184102.154	No	4426	184	1154
14	012	12	189092.272	No	3496	146	482
15	012	24	193143.779	No	8747	182	1289

† Not used in analysis.

**Table 4:** Dataset description for parameter selection study

Dataset No.	Parameter change
0	RingToIndex = 4
1	RingToIndex = 6
2	RingToIndex = 7
3	RingToIndex = 8
4	RingToIndex = 9
5	RingToIndex = 10
6	RingToIndex = 11
7	RingToIndex = 12
8	RingToIndex = 13
9	RingToIndex = 14
10	RingToIndex = 15
11	RingsToAnalyze = [4–9]
12	RingsToAnalyze = [4–12]
13	Confidence = 0.5
14	Confidence = 0.8
15	Confidence = 0.9
16	Lattice parameter†

† *a* = 2.927 Å, *b* = 2.927 Å, *c* = 4.675 Å, α = 90°, β = 90°, γ = 120°.

**Table 5:** Median and standard deviation in difference in strain between dataset 11 in Table 4 ▸ and reference reconstruction (Abs. = absolute)

	Abs. median	Abs. std dev.
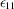	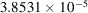	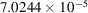
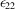	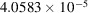	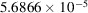
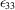	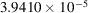	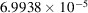

**Table 6:** Median and standard deviation in errors for lattice parameter (%) between dataset 16 in Table 4 ▸ and reference reconstruction

	Abs. median	Abs. std dev.
*a*	0.005432	0.016445
*b*	0.003553	0.010571
*c*	0.001401	0.007559

**Table 7:** Summary of FF-HEDM parameter sensitivity study For each parameter varied, the dominant impact on reconstruction quality and the resulting best practice are listed. All findings are quantified in the referenced figures and tables.

Parameter	Dominant impact	Best practice
RingToIndex	Completeness (especially for small grains); minimal impact on accuracy of matched grains [Fig. 4 ▸(*a*)]	Select the most intense, complete ring to maximize small-grain sensitivity. Ring multiplicity should be consistent with the chosen MinNrSols
Ring overlap (rings 8/9)	Initial peak misassignment is filtered out by crystallographic consistency during indexing and refinement (Fig. 5 ▸)	No user action required; the framework is intrinsically robust to such ambiguities
RingsToAnalyze range	Precision of position, grain size and strain. Excluding high-angle rings reduces 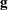 diversity and degrades refinement accuracy (Figs. 6 ▸, 7 ▸)	Include as many reliable rings as practical, especially high-angle rings, even when they have lower intensity
Confidence threshold	Primarily affects detection of small, low-completeness grains; well defined grains are stable across thresholds	Start with Confidence = 0.2–0.3; raise or lower based on whether completeness or certainty is prioritized
Initial lattice parameter	Refined values converge correctly for deliberate initial offsets, with median error smaller than the input deviation (Fig. 8 ▸)	No special handling required; the framework is robust to reasonable initial guesses

**Table 8:** Per-stage convergence of the decoupled iterative refinement, averaged over 250 grains with 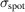 = 1 pixel noise Each stage progressively reduces the residual errors, with the dedicated strain fit (stage 3) producing the largest single improvement.

Stage	Free parameters	Position error (µm)	Omega error (°)	Internal angle (°)
1: Position	12	215.10	0.0752	0.0860
2: Orientation	9	184.32	0.0500	0.0621
3: Strain	6	130.78	0.0249	0.0400
4: Position	3	130.46	0.0249	0.0399

**Table 9:** Comparison of refinement strategies on 250 synthetic f.c.c. grains with known ground truth Lattice parameter errors are reported using the permutation-aware metric to account for cubic symmetry. The iterative method achieves both better accuracy and lower runtime despite using the same total computational budget.

	Position (µm)	Orientation (°)	Strain (%)	Refinement runtime (s)
No noise				
Iterative	2.04	0.083	0.001	23
All-at-once	1.37	0.083	0.190	35
				
Noisy ( 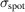 = 1 pixel)				
Iterative	33.91	0.085	0.011	23
All-at-once	38.87	0.083	0.252	35

**Table 10:** Dataset description for NF-HEDM

Dataset No.	Sample position 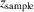 (µm)	Starting rotation angle 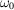 (°)	Displacement 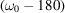 (°)	Rotation range (°)
0	0	−180.00	0	180.00
1	0	−179.90	0.1	180.00
2	−2	−179.90	0.1	180.00
3	−4	−179.90	0.1	180.00
4	0	−179.85	0.15	180.00
5	−2	−179.85	0.15	180.00
6	−4	−179.85	0.15	180.00
7	0	−179.80	0.2	180.00
8	−2	−179.80	0.2	180.00
9	−4	−179.80	0.2	180.00
10	0	−180.00	0	360.00

**Table 11:** Refined parameters of the experimental setup for the NF-HEDM experiment

	D1	D2	D3	D4	D5
*L* (µm)	4662.483	5667.281	6669.932	7671.419	8671.095
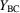 (pixels)	1030.317	1035.942	1042.048	1044.878	1056.553
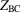 (pixels)	21.125	21.940	25.043	25.423	29.528
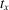 (°)	0.45486				
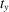 (°)	2.08128				
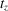 (°)	1.53305				

**Table 12:** NF displacement angles for different NF-HEDM scans

		Reconstruction computed displacement		
Dataset No.	Displacement angle 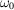 (°)	Mean (°)	Median (°)	Std dev. (°)
1	0.1	0.1147	0.1139	0.02896
2	0.1	0.1107	0.1099	0.02941
3	0.1	0.1109	0.1096	0.03111
4	0.15	0.1439	0.1433	0.03090
5	0.15	0.1408	0.1403	0.03095
6	0.15	0.1372	0.1368	0.02970
7	0.2	0.1754	0.1752	0.02951
8	0.2	0.1736	0.1732	0.02943
9	0.2	0.1711	0.1707	0.02852

## Data Availability

The raw FF-HEDM and NF-HEDM Ti-7 Al datasets used for the validation in this study are openly available in the Materials Data Facility at https://doi.org/10.18126/8487-hd29.

## Footnotes

1 This dictates that a minimum of three independent solutions are required to qualify a grain. See Part I of the series for more details.
